# *Akkermansia muciniphila*: A next-generation gut probiotic supporting neurorepair and functional recovery

**DOI:** 10.4103/NRR.NRR-D-25-00701

**Published:** 2025-09-29

**Authors:** Huiwen Yuan, Jingwei Shi, Chenlong Gu, Jinlong Yuan, Chenlei Huang, Xiaoning Li, Kailiang Zhou, Jianjun Qi

**Affiliations:** 1 Department of Laboratory Medicine, The First Affiliated Hospital of Wannan Medical College (Yijishan Hospital of Wannan Medical College), Wuhu, Anhui Province, China; 2 Department of Orthopedics, The Second Affiliated Hospital and Yuying Children’s Hospital of Wenzhou Medical University, Wenzhou, Zhejiang Province, China; 3 Cixi Biomedical Research Institute, Wenzhou Medical University, Ningbo, Zhejiang Province, China; 4 Department of Psychiatric Rehabilitation, Second People’s Hospital of Huizhou, Huizhou, Guangdong Province, China; 5 Department of Neurosurgery, The First Affiliated Hospital of Wannan Medical College (Yijishan Hospital of Wannan Medical College), Wuhu, Anhui Province, China

**Keywords:** *Akkermansia muciniphila*, brain–gut axis, clinical efficacy, gut microbiota, intestinal barrier, metabolic products, neurodegenerative disease, neuroinflammation, neurological disorders, neurotransmitter

## Abstract

The brain–gut axis is a bidirectional signal transduction system between the gastrointestinal tract and the central nervous system that integrates neural, endocrine, and immune functions. In recent years, the role of the intestinal flora in regulating neural function and affecting the progression of different neurological diseases has received increasing attention. *Akkermansia muciniphila* is a mucin-degrading bacterium of the intestinal flora present in the intestinal mucus layer that can regulate host immunity, the intestinal barrier and neuroimmune homeostasis. In recent years, a growing body of literature has suggested that *Akkermansia muciniphila* may play beneficial roles in nerve injury and regeneration by regulating brain–gut axis signalling. This review comprehensively summarizes the latest research results on the role of *Akkermansia muciniphila* in neurological diseases such as spinal cord injury, multiple sclerosis, Parkinson’s disease, and Alzheimer’s disease. The mechanisms by which *Akkermansia muciniphila* regulates inflammatory cytokines, neurotransmitters, and short-chain fatty acids are also highlighted. Various *Akkermansia muciniphila*-based interventions, such as those involving outer membrane proteins, extracellular vesicles, and pasteurized *Akkermansia muciniphila*, are discussed, and their therapeutic potential in restoring intestinal homeostasis, alleviating neuroinflammation, and supporting neuronal repair is explored. Although promising results from animal models have been reported, significant challenges remain in translating these findings into clinical practice and therapeutic applications. The differences in *Akkermansia muciniphila* colonization efficiency, host responses, and intervention strategies in different disease states limit the results of these studies. In addition, *Akkermansia muciniphila* may exhibit different mechanisms of action in acute and chronic neurodegenerative diseases, and thus more targeted mechanistic studies are needed. Despite these limitations, *Akkermansia muciniphila* represents a novel and potent pathway for the modulation of the brain–gut axis to support neural repair and functional recovery. By enhancing intestinal barrier integrity and regulating neuroimmunity, *Akkermansia muciniphila* has broad prospects as a microbial candidate for the treatment of central nervous system diseases. Future research should focus on optimizing the administration method and clinical trials to verify its efficacy, ultimately providing new treatment options in the field of neural regeneration and microbial therapy.

## Introduction

Neurological diseases and neuroinflammation, including Parkinson’s disease (PD), Alzheimer’s disease (AD) (Cheng et al., 2025; Martinez and Peplow, 2025), and migraine, are major causes of death and poor health worldwide (Samanta et al., 2024; Shi and Yong 2025). Current treatments for the nervous system generally include drug therapy, namely, neurotransmitter modulators (such as levodopa, paroxetine, and olanzapine; Dean and Standaert 2024; Zheng et al., 2024), central nervous system (CNS) stimulants/depressants (such as methylphenidate) (Shellenberg et al., 2020), neuroprotective agents/anti-inflammatory drugs (such as edaravone; Singh et al., 2024), surgical treatment, immunotherapy and targeted therapy, psychological therapy, and behavioural interventions (Lopes et al., 2022). However, these treatments have considerable drawbacks. Drug therapy is prone to side effects, surgical treatment is highly traumatic, and immunotherapy and targeted therapy carry the risk of long-term immunosuppression. Psychotherapy has limited effectiveness (García-González et al., 2024). Finding the right treatment method has become a tricky problem. In recent years, an increasing number of studies have shown that the gut microbiota, an important regulator, can achieve two-way signal regulation with the central nervous system through the gut–brain axis (Park et al., 2025; You et al., 2025), thereby slowing damage to and repairing the nervous system (Góralczyk-Bińkowska et al., 2022). The use of the gut microbiota for therapeutic purposes is a novel and emerging area of research, with certain specific gut bacterial strains currently serving as the focal point of ongoing studies.

The brain–gut axis is an intermediate regulator connecting the gastrointestinal tract and the central nervous system (Lee et al., 2023; Ju et al., 2025; Sun et al., 2025). It has bidirectional conduction and complex characteristics (Kraimi et al., 2024), including multiple components such as the vagus nerve, enteric nervous system, and endocrine pathways (Naufel et al., 2023). Specifically, the gut microbiota secretes metabolic products such as short-chain fatty acids (SCFAs), neurotransmitters, inflammatory factors, and other signaling molecules, which ultimately regulate the levels of inflammatory factors, neurotransmitter balance, metabolic balance, blood–brain barrier stability, and immunity in the central nervous system (Fan and Pedersen, 2021; Dicks, 2022). Recent studies have shown that the brain–gut axis plays a key regulatory role in emotions, cognition (Toader et al., 2024), and neural regeneration (Merlo et al., 2024). Therefore, identifying specific gut bacterial strains that can alleviate neurological diseases through the important medium of the gut–brain axis has become a key issue that needs to be addressed.

**Figure 1:**
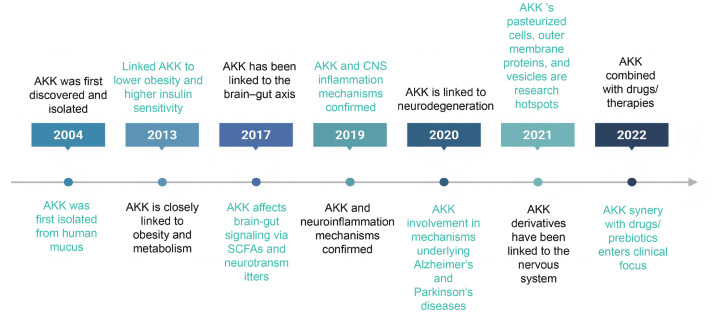
AKK and the timeline of nervous system development. Created with BioRender.com. AD: Alzheimer’s disease; AKK: *Akkermansia muciniphila*; CNS: central nervous system; SCFAs: short-chain fatty acids.

*Akkermansia muciniphila* (AKK) is an oval, nonmotile, strictly anaerobic, gram-negative bacterium first isolated from human feces in 2004 (Zhou and Zhang, 2019; Rodrigues et al., 2022; **Figure 1**). AKK resides within the mucus layer of the human intestinal tract and uses mucin secreted by intestinal epithelial cells as its main carbon source. By degrading mucin, AKK produces SCFAs, which are essential for intestinal health because they regulate the acid-base balance in the gut (Kim et al., 2022). AKK can briefly colonize the mucous layer of the intestine, and the reason for this short-term colonization may be that AKK possesses various genes and proteins related to mucin and surface adhesion, which may support short-term biofilm formation and the attachment of AKK to the mucosal surface (Mulhall et al., 2020; Trastoy et al., 2020). These structures can help AKK adhere to and enhance the integrity of the intestinal barrier, but whether they can form a lasting biofilm still requires further research. Additionally, AKK is unable to form spores, and thus its survival relies on the ability to use resources from the external environment rather than being able to colonize based on its own favorable resources (Zeng et al., 2025). Finally, we speculate that AKK may have a metabolically complementary relationship with other gut microbiota, and that the monosaccharides released after its degradation of mucin can be used by other bacteria. Although AKK comprises only approximately 3% of the intestinal microbiota, it plays a critical role in maintaining the intestinal barrier (Liu et al., 2022). It increases the thickness of the mucus layer and improves barrier function, preventing pathogenic microorganisms from crossing the intestinal epithelium (Geerlings et al., 2021). AKK has significant anti-inflammatory effects, especially in diseases associated with intestinal inflammation, such as inflammatory bowel disease (Hasani et al., 2021). Changes in AKK abundance affect gut health, with reduced levels linked to metabolic diseases, including obesity and diabetes, as well as chronic inflammation (Mo et al., 2024). Recent studies have focused on the association between AKK abundance and metabolic disorders, describing correlations with the development of obesity (Zheng et al., 2022) and type 2 diabetes (Effendi et al., 2022). For example, reduced AKK levels in patients with type II diabetes are correlated with insulin resistance, whereas supplementation with AKK improves postprandial blood glucose levels (Zhang et al., 2021).

In addition to its role in metabolic diseases, AKK is also implicated in cardiovascular diseases (Kobyliak et al., 2022) and liver disorders (Perraudeau et al., 2020). It ameliorates hypertension by producing SCFAs that strengthen the gut barrier, reduce the production of inflammatory factors, and modulate inflammatory signaling pathways (He et al., 2022a, b). Furthermore, AKK reportedly attenuates metabolic dysfunction-associated fatty liver disease by regulating L-aspartic acid metabolism, activating the LKB1-AMP-activated protein kinase (AMPK) signaling pathway, decreasing oxidative stress in the liver, and lowering the levels of inflammatory cytokines (Sparfel et al., 2024). These findings collectively indicate that AKK has promising potential to improve a wide range of systemic diseases (Zhang et al., 2024a). Moreover, its therapeutic effects extend to the modulation of inflammatory responses (Lakshmanan et al., 2022) and the enhancement of metabolic homeostasis (Rao et al., 2021). Additionally, AKK considerably influences the nervous system by modulating the brain–gut axis through multiple mechanisms. These mechanisms include maintaining the integrity of the intestinal barrier (Palmnäs-Bédard et al., 2022), regulating the levels of inflammatory factors (Xu et al., 2023), affecting mood and functions (Li et al., 2023), and modulating the concentrations of SCFAs (Wang et al., 2025d). Although research on AKK in neurological diseases is still in the early stages, related studies have revealed an important role for AKK in the repair of neuroinflammation and central nervous system damage, which requires further exploration.

Therefore, this review aims to explore the relationships between AKK and neurological diseases, nervous system inflammation, and repair and damage. It summarizes the specific mechanisms of action of the brain–gut axis mediated by AKK in the nervous system, such as SCFAs and neurotransmitters, to demonstrate that AKK has a positive therapeutic effect on the nervous system. Additionally, this review summarizes the effects of AKK on inflammation levels and nerve regeneration, analyzes its roles in PD, AD, anxiety, and depression, and examines the disadvantages of AKK, including challenges in clinical translation, safety concerns, individual variability, and whether different strains exhibit similar activity. We will also look toward future research, discussing whether we should adopt new drug delivery methods, combine targeted drugs, or intervene through diet. All of these aspects will be explored to provide a rational basis for the future treatment of neurological diseases.

## Search Strategy

This review explores how AKK affects the treatment of neurological diseases and the injury and repair of neuroinflammation through the brain–gut axis axis. More than 96% of the cited articles were published in English, and over 70% were published in the past 5 years. The literature collection period ranges from October 2024 to January 2025. The search engines used were PubMed, CNKI, and Web of Science databases. Peer-reviewed original articles and review articles that focused on the role of AKK in nerve injury repair and regeneration were included. The exclusion criteria consisted of non-English or non-Chinese publications, articles not directly related to the brain–gut axis axis or neurobiology, duplicate studies, and conference abstracts without sufficient data. The following keywords were used to search the relevant literature: *Akkermansia muciniphila*, gut microbiota, neuroinflammation, nerve injury, neurodegenerative diseases, the brain–gut axis, neurotransmitters, and SCFAs. Ultimately, 252 articles were included in this review and analysis. All years were considered in the search.

## Mechanism of Action of *Akkermansia Muciniphila* in the Brain–Gut Axis

### *Akkermansia muciniphila* protects the nervous system from damage by maintaining the integrity of the gut barrier

**Figure 2:**
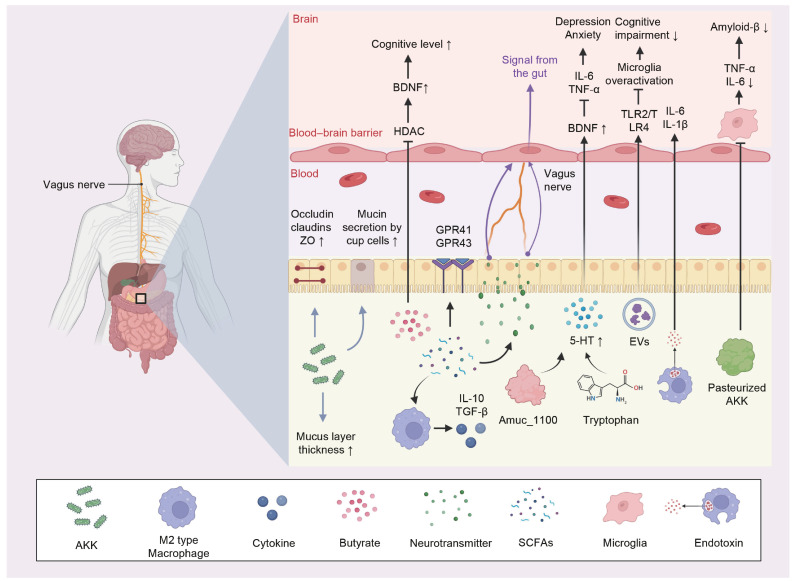
Mechanism of action of AKK and its regulation of the brain‒gut axis. AKK maintains the integrity of intestinal barrier function. It upregulates tight junction proteins (occludin, claudins, and ZO proteins) by interacting with intestinal epithelial cells. Upregulated tight junction proteins enhance intestinal barrier function and reduce the entry of harmful substances, pathogenic bacteria, and toxins into the bloodstream. Additionally, AKK stimulates the secretion of mucin from goblet cells, which increases the thickness of the intestinal mucus layer. SCFAs modulate the effects of the brain–gut axis on the nervous system. AKK increases the production of anti-inflammatory cytokines (such as IL-10) and decreases the production of pro-inflammatory cytokines (including IL-6 and TNF-α) through the production of SCFAs. This process activates G protein-coupled receptors 41 and 43 in the intestine, promoting the polarization of macrophages toward the M2 type and attenuating inflammatory responses in the nervous system. Moreover, SCFAs stimulate the vagus nerve, an important bridge between the gut and the brain, transmitting signals that regulate the function of the nervous system. Additionally, the butyrate component of SCFAs can inhibit histone deacetylases, increasing histone acetylation, promoting the expression of genes related to nerves and memory (such as BDNF), and improving cognitive and learning functions, leading to protective effects on the nervous system. The effects of the outer membrane protein Amuc_1100 on neurological mood via the brain–gut axis have also been studied. Amuc_1100 regulates the levels of the neurotransmitter serotonin and improves mood regulation by upregulating BDNF expression and enhancing anti-inflammatory effects to reduce inflammatory responses in the brain–gut axis. AKK-derived EVs attenuate neurologically relevant cognitive deficits through the brain–gut axis. These EVs inhibit microglial activation through two signaling pathways, TLR2 and TLR4, which reduce neuroinflammation and postoperative cognitive dysfunction, thereby protecting normal brain function. Bachmann-inactivated AKK attenuates neurological disorders through multiple mechanisms. It inhibits neuroinflammatory responses via the brain–gut axis, which in turn reduces microglial activation and decreases oxidative stress, resulting in neuroprotection and improvements in cognitive and behavioral performance, such as memory and anxiety. Endotoxin release can affect the nervous system; lipopolysaccharides enter the bloodstream by increasing intestinal permeability, triggering an inflammatory response. These endotoxins can then enter the brain through the blood-brain barrier, where they activate microglia to release inflammatory factors, triggering neuroinflammation, and exacerbating neurodegenerative diseases. Created with BioRender.com. 5-HT: 5-Hydroxytryptamine; AKK: *Akkermansia muciniphila*; BDNF: brain-derived neurotrophic factor; EVs: extracellular vesicles; GRP41: G protein-coupled receptor 41; GRP43: G protein-coupled receptor 43; HDACs: histone deacetylases; IL-10: interleukin-10; IL-1β: interleukin -1β; IL-6: interleukin-6; SCFAs: short-chain fatty acids; TNF-α: tumour necrosis factor alpha; ZO: zonula occludens.

The intestinal barrier is a multifaceted structure comprising four integral components: the physical barrier, chemical barrier, immune barrier, and microbiological barrier (Martel et al., 2022). Importantly, the physical barrier, which is predominantly composed of the intestinal mucus layer and tight junction proteins, functions as the primary defense mechanism against luminal pathogens and toxins (Untersmayr et al., 2022). Within this broader analytical framework, the mucus layer, synthesized from mucins secreted by intestinal goblet cells, forms a protective coating over the epithelial surface, thereby preventing direct interactions between harmful agents and epithelial cells (Zhai et al., 2019a; Gustafsson and Johansson, 2022; Luis and Hansson, 2023). AKK, an obligate mucin-degrading bacterium, plays a critical role in sustaining the structural and functional integrity of the mucus layer through the enzymatic breakdown and recycling of mucins (Hasani et al., 2021; Zhao et al., 2023). Emerging evidence suggests the therapeutic potential of AKK in restoring intestinal barrier homeostasis. For example, the administration of pasteurized AKK or its derived protein, Amuc_1100, appears to mitigate antibiotic-associated diarrhea (AAD) by upregulating the expression of tight junction proteins, including zonula occludens-1 (ZO-1), occludin, claudin-4, and mucin 2 (Muc2), thereby apparently attenuating colonic damage and diarrhea symptoms (Liu et al., 2024; **Figure 2**). Similarly, in a murine model of gestational diabetes, AKK supplementation tends to ameliorate insulin resistance, which is indicated by reductions in fasting blood glucose levels, insulin levels, and insulin resistance (IR), while seemingly increasing hepatic Akt phosphorylation. In this broader analytical context, AKK appears to decrease systemic levels of pro-inflammatory cytokines (tumor necrosis factor-alpha [TNF-α] and interleukin-1 beta [IL-1β]) and lipopolysaccharide (LPS) while ostensibly upregulating the expression of tight junction proteins (ZO-1 and occludin). The apparent reinforcement of intestinal barrier function is especially noteworthy (Yang et al., 2024). These findings suggest that AKK has the capacity to increase tight junction protein expression, maintain mucus layer integrity, and prevent the translocation of luminal antigens into the systemic circulation. Notably, a diminished abundance of AKK tends to be associated with reduced mucin secretion, thinning of the mucus layer, and compromised barrier function, potentially contributing to the pathogenesis of various gastrointestinal and systemic disorders. Moreover, AKK appears to modulate neurological function through the brain–gut axis, with its depletion seemingly implicated in striatal damage and the onset of neurological disorders (Bi et al., 2023; Zhang and Wang, 2023; Panzetta and Valdivia, 2024).

AKK also exerts profound effects on neurobehavioral regulation. For example, one study revealed a correlation between AKK and the dysregulation of the mesolimbic reward system in obese mice (Huwart et al., 2022). Obese mice exhibited an attenuated preference for palatable foods, accompanied by reduced motivation for reward-seeking behaviors and downregulated expression of dopamine (DA) receptors. This pattern is generally indicative of impaired dopaminergic signaling and is also associated with elevated expression of pro-inflammatory genes, diminished tight junction protein levels in the striatum, and compromised blood–brain barrier integrity (Huwart et al., 2022). Notably, AKK intervention reversed many of these pathological alterations, increasing DA receptor expression, attenuating neuroinflammation, upregulating tight junction proteins, and ultimately restoring reward-seeking behavior (Huwart et al., 2022). Additionally, the findings from Huwart et al. (2022) suggests that in Negr1 gene knockout mice, the abundance of AKK tends to be reduced. Transcriptomic sequencing has shown that AKK regulates the expression of immune-related genes in the intestine while also influencing the expression of mitochondria-related genes in the brain (Hwang et al., 2025). Subsequent behavioral tests revealed that these mice exhibited depression-like behaviors, supporting the occurrence of neuronal atrophy (Hwang et al., 2025). Given the complexity of these theoretical relationships, AKK appears to be critically important for maintaining neuronal integrity (Hwang et al., 2025). A randomized controlled trial compared 43 healthy individuals with 43 patients diagnosed with major depressive disorder (Mörkl et al., 2025). After 3 months of probiotic supplementation, the abundance of AKK in the gut increased, suggesting that AKK tends to maintain the stability of intestinal epithelial cells by regulating their proliferation, differentiation, and apoptosis (Mörkl et al., 2025). Within this broader analytical framework, AKK, as a probiotic, appears to increase mucus layer thickness and regulate microbial balance; these mechanisms may ultimately improve depressive symptoms in many patients (Mörkl et al., 2025). Given the complexity of these theoretical relationships, these findings warrant further interpretive consideration (Herath et al., 2025).

Individuals with autism spectrum disorder (ASD) often exhibit impaired intestinal barrier function. An investigation into whether the Neuroligin-3 gene R451C variant associated with ASD affects the intestinal mucus layer and gut microbiota in mice used techniques such as fluorescence in situ hybridization and immunofluorescence staining to confirm the presence of the genetic variant (Herath et al., 2025). The results revealed changes in mucus density near the distal ileal epithelium, including increased densities of Firmicutes and AKK, reduced levels of Bacteroides, and overall modulation of the spatial microbial distribution. This led to the conclusion that this mutation influences both the thickness of the mucus layer and the gut microbiota structure, thereby highlighting the profound influence of neurodevelopmental disorders on gut microbial ecosystems (Herath et al., 2025). Recent research has indicated that AKK may ameliorate striatal dysregulation induced by metabolic dysfunction (Gao et al., 2024). Notably, AKK and its extracellular vesicles (EVs) also have potential for alleviating postoperative cognitive dysfunction (Gao et al., 2024). In murine models, this condition is frequently associated with reduced fecal AKK abundance, elevated systemic inflammation, and impaired blood–brain barrier permeability (Gao et al., 2024). Mechanistically, AKK supplementation upregulates tight junction proteins (e.g., occludin and ZO-1) in intestinal epithelial cells, improves cognitive performance in behavioral tests, and suppresses neuroinflammation by inhibiting the TLR2/4 signaling pathway (Gao et al., 2024). Collectively, these effects suggest that AKK plays a role in restoring blood–brain barrier integrity (Gao et al., 2024). These studies highlight the neuroprotective role of AKK through the reinforcement of intestinal barrier function and attenuation of systemic inflammation (Huwart et al., 2022; Gao et al., 2024; Herath et al., 2025; Hwang et al., 2025; Mörkl et al., 2025). For example, AKK mitigates cognitive deficits in a murine model of AD by reducing circulating endotoxin and triglyceride levels, preserving intestinal barrier integrity, and decreasing hippocampal amyloid-β plaque deposition, thereby improving cognitive outcomes (Ou et al., 2020).

Levodopa can be used to treat early-stage PD. A study compared the fecal microbiota of 31 patients with early-stage PD who had not received levodopa treatment with that of 28 healthy individuals (Bedarf et al., 2017). Metagenomic shotgun sequencing revealed increased abundance of the *Verrucomicrobiaceae* family, including AKK, and the *Firmicutes phylum*. Microbial metabolism includes β-glucuronic acid and tryptophan metabolism (Bedarf et al., 2017). These findings suggest that AKK can restore the intestinal mucus layer and epithelial cells in high-fat diet-fed mice, improving intestinal barrier function. Additionally, AKK may break down the mucosal mucin layer (Bedarf et al., 2017). Damage to intestinal barrier function can allow harmful substances to more easily enter the bloodstream, potentially affecting the central nervous system (Bedarf et al., 2017). Additionally, a traditional Chinese medicine known as *Danggui Sini* has the potential to relieve sciatic nerve pain through a reduction in chronic constrictive damage in mice (Di et al., 2024). Within this broader analytical framework, the abundance of AKK appears to increase, and evidence suggests that the levels of inflammatory factors in rats tend to normalize (Di et al., 2024). Given the complexity of these theoretical relationships, nerve repair tends to be promoted by a decrease in inflammatory factors, which seemingly contributes to a treatment approach for sciatic nerve pain (Di et al., 2024). Nevertheless, current methodologies for assessing intestinal barrier integrity remain relatively rudimentary (González-Mariscal et al., 2011). While techniques such as qPCR and immunofluorescence staining have been used to evaluate gene and protein expression, more sophisticated approaches, including Western blot analysis and immunoelectron microscopy, could provide higher-resolution insights into the dynamics of tight junction proteins (González-Mariscal et al., 2011). Indirect measures such as LPS quantification could be supplemented with direct techniques, such as fluorescein isothiocyanate dextran permeability assays, to quantitatively assess intestinal barrier integrity. For example, in a study of the blood-brain barrier, Evans Blue can be combined with high-molecular-weight FITC-dextran and supplemented with fluorescence quantitative analysis to enable semi-quantitative functional localization (Xu et al., 2019b). Furthermore, the translocation of fluorescein isothiocyanate-dextran into the systemic circulation following barrier disruption appears to support a robust and largely quantifiable metric, thereby advancing the precision and reliability of intestinal barrier.

### Neurotransmitter regulation by *Akkermansia muciniphila* in the brain–gut axis

**Figure 3:**
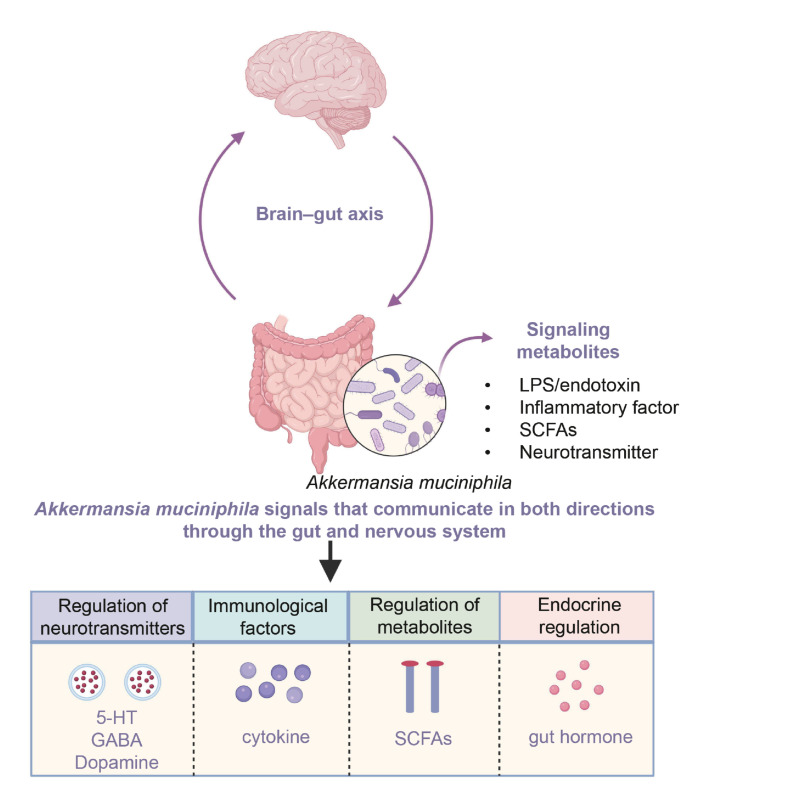
AKK communicates bidirectionally through the gut and nervous system. AKK affects the synthesis and metabolism of neurotransmitters, especially the levels of 5-HT, DA, and GABA, by regulating the balance of the gut microbiota. These neurotransmitters play roles in mood, cognition, and behavior. Increasing the AKK levels helps improve mood disorders such as anxiety and depression and contributes to cognitive enhancement. AKK reduces neuroinflammation by enhancing intestinal barrier integrity and limiting the entry of harmful substances and proinflammatory factors. It modulates intestinal immune responses and prevents microglial overactivation. AKK exerts antineuroinflammatory effects by regulating inflammatory cytokine production to reduce neuronal damage and promote recovery, thus regulating microglial activation. Through the production of SCFAs, AKK exerts anti-inflammatory, neuroprotective, and cognitive effects. These SCFAs provide energy to intestinal cells and can cross the blood–brain barrier to influence brain function. Additionally, AKK regulates metabolic health by modulating the secretion of intestinal hormones such as GLP-1 and insulin, further contributing to the attenuation of neuroinflammation. Created with BioRender.com. 5-HT: 5-Hydroxytryptamine; AKK: *Akkermansia muciniphila*; DA: dopamine; GABA: gamma-aminobutyric acid; GLP-1: glucagon-like peptide 1; LPS: lipopolysaccharide; SCFAs: short-chain fatty acids.

The gut microbiota, a critical component of the brain–gut axis, exerts profound effects on brain function through multiple mechanisms. These findings suggest that one key mechanism involves the production and secretion of microbe-derived neurotransmitters (Qu et al., 2024). Neurotransmitters, within this broader analytical framework, are endogenous chemical messengers that facilitate synaptic transmission by apparently relaying signals between neurons, thereby modulating neuronal excitability and synaptic plasticity (Qu et al., 2024). These microbe-derived neurotransmitters can influence neural activity, either by exerting excitatory or inhibitory effects, and they have a pivotal role in regulating interneuronal communication and brain function (Chen et al., 2021). Neurotransmitters are typically classified as either excitatory or inhibitory based on their functions (Strandwitz 2018; Ayala-Lopez and Watts 2021; Nimgampalle et al., 2023). Serotonin (5-HT), DA, gamma-aminobutyric acid (GABA), and glutamate are important neurotransmitters in the gut–brain axis, playing key roles in both the peripheral nervous system and the CNS (Qu et al., 2024). Notably, the literature suggests that gut microbes can influence the production and release of neurotransmitters (Dinan and Cryan, 2017; Dicks, 2022). Evidence indicates that gut microbes use a variety of neurotransmitters for two-way communication with the CNS (Dinan and Cryan, 2017). Within this broader analytical framework, these neurotransmitters appear to function as signaling molecules to the brain via afferent vagal fibers. In turn, these fibers seem to transmit signals to enteric nerve cells in the intestinal wall and to the mucosal immune system via efferent nerve fibers (Dicks, 2022). These neurotransmitters and intestinal microorganisms play crucial roles in maintaining intestinal immune homeostasis and balance, which, when disrupted, may indicate the potential to cause gastrointestinal disorders. Given the complexity of these theoretical relationships, such disruptions could ultimately affect the function of the nervous system and potentially lead to neurodegenerative diseases (Dicks, 2022). Additionally, the article by Strandwitz (2018) summarizes how intestinal microorganisms produce and regulate a variety of key neurotransmitters, including dopamine, norepinephrine, 5-HT, and gamma-aminobutyric acid. These microorganisms not only synthesize neurotransmitters but also influence the host’s CNS function in animal models, revealing the bidirectional communication mechanism between intestinal microorganisms and the brain (Strandwitz, 2018). AKK is also involved in regulating the brain-gut axis by affecting the synthesis and metabolism of neurotransmitters (5-HT, GABA, and DA; Ding et al., 2021; Gu et al., 2021; **Figure 3**). A detailed discussion of the different neurotransmitters is provided below.

#### Effects of Akkermansia muciniphila on 5-hydroxytryptamine

The majority of 5-HT is synthesized in the gut, and its precursor is tryptophan. Tryptophan metabolism involves several enzymes (De Deurwaerdere and Di Giovanni 2021; Pourhamzeh et al., 2022). First, tryptophan hydroxylase is the rate-limiting enzyme for 5-HT synthesis and is responsible for the conversion of tryptophan to 5-hydroxytryptophan, which is subsequently converted to 5-HT by the action of aromatic L-amino acid decarboxylase (Kałużna-Czaplińska et al., 2019). In contrast, AKK indirectly affects 5-HT synthesis by promoting tryptophan metabolism and regulating intestinal mucosal health (Yaghoubfar et al., 2020). Sun et al. (2023) indicated that AKK and its outer membrane protein Amuc_1100 reduced depressive and anxiety-like behaviors in mice, although the specific mechanisms underlying this effect remain unclear. Following antibiotic-induced gut microbiota dysbiosis, most mice exhibited increased anxiety- and depression-like behaviors, which are subsequently alleviated by AKK supplementation through the reduced expression of genes associated with these conditions and alterations in neurotransmitter levels, particularly 5-HT levels, in the hippocampus and cortical tissue (Sun et al., 2023). These findings are particularly noteworthy, as AKK appears to broadly mitigate depressive and anxiety-like behaviours by modulating 5-HT levels. In one study, the administration of AKK and its EVs increased 5-HT levels in the colon and hippocampus while reducing 5-HT levels (Yaghoubfar et al., 2020). These measures also altered the expression of serotonergic genes, highlighting the regulatory role of AKK in the serotonergic system through the gut‒brain axis (Yaghoubfar et al., 2020). Similarly, two approaches, chronic alcohol exposure and chronic unpredictable mild stress, have been used to induce depressive behaviours in mice, with tests such as the forced swimming test, tail suspension test, and sucrose preference test revealing both despair-like behaviours and anhedonia, along with a substantial decrease in 5-HT levels in the gut and brain (Guo et al., 2024). Following AKK intervention, mice exhibited a noticeable increase in sucrose preference, a reduction in resting time, elevated 5-HT levels, and decreased cFos expression in enteric nerves. This was hypothesized to modulate brain–gut signaling by inhibiting enteric nerve activation, potentially affecting the bidirectional transmission of brain–gut signals (Guo et al., 2024). A study reported that AKK appears to modulate 5-HT via the gut-liver-brain axis, thereby alleviating neurological cognitive impairment. The study also identified associations between hepatic encephalopathy, a complication of cirrhosis and cognitive decline (Kang et al., 2024a). 16S rRNA gene sequencing revealed that AKK levels were reduced in most patients with hepatic encephalopathy. In a mouse model mimicking liver injury, reduced BDNF and 5-HT levels, increased levels of inflammatory markers, and cognitive impairment were observed. However, the AKK intervention seemingly reversed these changes by restoring cognitive performance, lowering pro-inflammatory factors, and increasing BDNF and 5-HT levels (Kang et al., 2024a). Another study reported that supplementation with AKK improved alcohol-related depression, reduced serum LPS levels, lowered serum levels of inflammatory factors, and increased hippocampal levels of 5-HT (Guo et al., 2022). By increasing the expression of limiting enzymes, such as tryptophan hydroxylase 1 in the intestine and TPH2 in the central nervous system, AKK promotes the synthesis of 5-HT (Guo et al., 2022). A recent *in vitro* study showed that AKK-derived extracellular membrane vesicles can upregulate the expression of TPH1 and TPH2, ultimately increasing the activity of 5-HT (Yaghoubfar et al., 2020). This research used human Caco-2 intestinal epithelial cells and demonstrated that AKK can influence the expression of neurotransmitters by regulating the activity of related enzymes (Yaghoubfar et al., 2020). The findings of this work illustrate that supplementation with AKK affects the nervous system by modulating 5-HT levels in the gut, thereby improving mood disorders such as anxiety and depression.

#### Effects of Akkermansia muciniphila on gamma-aminobutyric acid

GABA is the main inhibitory neurotransmitter in the brain, reducing neuronal overexcitation, regulating brain activity, helping relieve anxiety, improving sleep, and playing an important role in mood and cognitive functioning (Duman et al., 2019; Sears and Hewett, 2021; Nieto et al., 2022). By binding to its receptors (GABA-A and GABA-B), GABA inhibits the transmission of neuronal signals and modulates neuronal excitability, which is crucial for maintaining a balanced brain state (Sivilotti and Nistri, 1991). GABA is strongly associated with neurological disorders such as depression, anxiety, and epilepsy (Duman et al., 2019). Li et al. (2022) noted differences in the brain–gut axis between patients with bipolar disorder and healthy controls, as revealed through fecal macrogenomics, serum metabolomics, and neuroimaging. These findings indicate that the levels of the microbially produced neuroactive metabolite GABA correlate with the abundance of AKK. Within this broader analytical framework, the microbial production of GABA is ostensibly influenced by specific gut microbes, such as AKK, and appears to be linked to brain network dysfunction, difficulties in emotion regulation, and cognitive dysfunction in the majority of patients with bipolar disorder. This suggests that AKK may contribute to regulating mood and cognitive functions by modulating GABA levels in the gut and brain (Li et al., 2022). Additionally, the literature indicates that the combined action of intestinal lactate and AKK can upregulate Tph1 and inhibit IDO1, shifting tryptophan metabolism towards 5-HT production. In a mouse model of anxiety, the combination of lactate and AKK remarkably increased 5-HT levels and alleviated anxiety behaviors (Pan et al., 2025).

A ketogenic diet (KD) is often used to treat epilepsy. Olson et al. (2018) suggested that gut flora plays an important role in antiepileptic therapy. Their experiment indicated that seizures in mice, stimulated with electrical currents and followed by the use of antibiotics to deplete the intestinal flora, yielded particularly considerable findings. Specifically, they found that a KD was ostensibly unable to increase the seizure threshold after the removal of gut flora, suggesting a necessary role for gut microbes in the antiepileptic effect of a KD (Olson et al., 2018). Within this broader analytical framework, subsequent experiments involving the transplantation of AKK and *Parabacteroides* into germ-free or antibiotic-treated mice seemingly led to attenuated epileptic behaviors and increased serum GABA/glutamate ratios after a KD. AKK in the gut appears to reduce epileptic seizure thresholds by increasing neurotransmitter levels (specifically GABA), thereby presumably reducing seizure frequency in most cases (Olson et al., 2018). A recent experiment investigated whether AKK has the ability to produce GABA. Based on the results of proteomic and metabolite analyses, AKK can typically produce GABA under acidic conditions and in the presence of glutamate or glutamine, which supports its role in the brain–gut axis and in regulating neurological health (Konstanti et al., 2024). These findings suggest that AKK not only plays a role in regulating neurotransmitter levels but may also contribute to maintaining brain homeostasis by influencing GABA levels in the intestine. The results indicate that AKK apparently prevents neurological disorders indirectly by regulating GABA levels in the gut to a predominantly healthy level, which may have far-reaching implications for the management of neuropsychiatric diseases, epilepsy, and mood disorders (Olson et al., 2018; Li et al., 2022; Konstanti et al., 2024). The relationship between gut bacteria and neural function is particularly noteworthy, given the complexity of these theoretical relationships. Can AKK regulate the balance of GABA/glutamate by adjusting the activity of certain enzymes? Although its specific mechanism has not yet been thoroughly studied, evidence suggests that the presence of AKK generally indicates a positive correlation with increased GABA levels in several preclinical anxiety and depression models (Maftoon et al., 2024). Further exploration is needed to establish more definitive connections. Regarding the downstream effects of the pathways regulated by these neurotransmitters, they have profound effects on the signaling pathways of nerve conduction (Maftoon et al., 2024). One finding from an animal experiment demonstrated that AKK supplementation can substantially reduce anxiety-like and depression-like behaviors in the majority of cases, although the precise mechanisms underlying these apparent effects warrant further interpretive consideration (Jiang et al., 2024). These findings also indicate that AKK ultimately produces positive results by regulating neurotransmitter levels. Therefore, AKK, a beneficial gut bacterium that can regulate the nervous system, greatly improves neural function.

#### Effects of Akkermansia muciniphila on dopamine

DA, a neurotransmitter, plays important roles in regulating mood, controlling movement, regulating learning and memory, and regulating endocrine function (Lakard et al., 2021). DA is synthesized mainly in the substantia nigra and ventral tegmental area of the brain and affects neuronal activity by binding to specific receptors, such as D1 and D2 receptors, which are critical for regulating both excitatory and inhibitory signals in the brain (Klein et al., 2019; Liu and Kaeser, 2019; Salvatore, 2024). DA is involved in a wide range of processes, including the reward system, motor control, and the regulation of emotional responses (Klein et al., 2019). In general, DA helps maintain normal motor, emotional, and perceptual activity. However, evidence suggests that dysregulation of DA is associated with various mental illnesses (Dudzik et al., 2024). For example, DA deficiency is a hallmark of Parkinson’s disease (PD), while excessive DA release has been implicated in schizophrenia and chorea. Dysfunction in DA signaling is also associated with mood disorders such as depression and anxiety, as well as addiction (Howes et al., 2024).

A study has indicated that AKK increases serum DA concentrations and effectively improves depression-like behaviors induced by chronic restraint stress in mice, suggesting that AKK may help alleviate stress-induced alterations in neurotransmitter balance, particularly dopamine. Serological experiments further showed that AKK substantially reversed the reduction in DA levels caused by chronic stress. In addition, AKK metabolites, including SCFAs such as butyrate, positively influence DA concentrations, suggesting that AKK modulates DA levels via gut–brain interactions (Ding et al., 2021). These findings underscore the potential of AKK to alleviate neurological depressive behaviors in mice by regulating DA levels (Ding et al., 2021). 6-OHDA is a neurotoxin that is structurally similar to DA (Guimarães et al., 2021). Functionally, it acts on dopaminergic neurons, ultimately leading to their death (Guimarães et al., 2021). A previous study revealed that after supplementation with AKK, the expression of 6-OHDA was upregulated, damage to dopaminergic neurons in the substantia nigra was reduced, motor function in rats was restored, inflammation levels were reduced, and neuronal death was remarkably decreased (Xu et al., 2025). All these studies collectively confirm that intestinal AKK modulates DA levels, which, in turn, affects neurological disorders, particularly mood and motor-related dysfunction (Ding et al., 2021; Guimarães et al., 2021; Dudzik et al., 2024; Xu et al., 2025). By influencing DA concentrations in the gut and brain, AKK might be a valuable tool for managing a range of neuropsychiatric disorders, such as depression, PD, and conditions associated with DA imbalance. By summarizing the three neurotransmitters described above (5-HT, GABA, and DA), we identified several shortcomings in the existing experiments. For example, an experiment in which DA receptor antagonists were used to treat patients with tic disorders ultimately indicated that AKK has potential therapeutic effects on intestinal metabolism, inflammatory bowel disease, and autism (Xi et al., 2021). However, the sample for this experiment was derived from a small, homogeneous population, and a larger population was not screened (Xi et al., 2021). Additionally, the metabolic function of the gut microbiota predicted by HUMAnN3 in this study lacked sufficient information on metabolite levels, which resulted in the absence of some necessary evidence (Xi et al., 2021). A study has shown that the long-term use of vancomycin can lead to changes in the gut microbiota in the body, particularly a considerable increase in AKK abundance (Zhang et al., 2022b). Although the quantity increased, the level of DA in the body decreased. Therefore, the increase in AKK abundance due to antibiotic interference may not necessarily have a positive effect on neural function (Zhang et al., 2022b). In addition, previous studies of the effects of AKK on neurotransmitter release are indirect analyses, primarily relying on corresponding behavioral tests and gene expression levels to determine changes in the mood and cognitive levels of the animals and subsequently observing alterations in neurotransmitter levels (Ding et al., 2021; Pan et al., 2025).

Another experiment focused on Chinese shooting athletes revealed that lactic acid and AKK in the gut can work together to regulate tryptophan metabolism, increase 5-HT activity, and ultimately alleviate anxiety (Pan et al., 2025). The initial findings of this study indicated that lactic acid can downregulate the expression of indoleamine 2,3-dioxygenase 1 (IDO1), altering the tryptophan pathway and shifting tryptophan metabolism from the kynurenine pathway to the 5-HT pathway, ultimately increasing 5-HT synthesis (Pan et al., 2025). Additionally, lactic acid can upregulate the expression of MCT1, a protein that promotes lactic acid transport between cells, in the mouse colon, thereby regulating tryptophan metabolism (Pan et al., 2025). However, the specific mechanisms underlying the interactions between metabolites and the gut microbiota, as well as which active component between AKK and lactic acid plays a dominant role, remain to be elucidated (Pan et al., 2025).

A study has shown that AKK has antidepressant effects in a mouse model of depression induced by chronic stress, with one key mechanism being that AKK can restore DA levels disrupted by chronic stress (Ding et al., 2021). As a neurotransmitter closely related to mood regulation, DA levels decrease considerably during depression, but after AKK intervention, DA levels are increased, indicating that AKK has a positive effect on the nervous system (Ding et al., 2021). However, these studies lack direct quantitative measurements of neurotransmitter levels (Ding et al., 2021; Pan et al., 2025). For example, one experiment measured lipid metabolites in feces, the hippocampus, and serum and inferred neurotransmitter expression levels based on the levels of these metabolites (Tian et al., 2022). Another study analyzed human epithelial colorectal cancer cells by extracting total RNA and then performing quantitative polymerase chain reaction to assess changes in gene expression levels, ultimately indirectly reflecting 5-HT levels to determine neurotransmitter content (Yaghoubfar et al., 2020). We seem to be able to detect neurotransmitter levels in the brain using methods such as liquid chromatography–mass spectrometry and to assess neurotransmitter levels through behavioral tests, which allows for a more comprehensive and ostensibly precise evaluation of the results. Furthermore, the following question appears particularly important: is AKK the only probiotic that promotes neurotransmitter synthesis? A study by Proano et al. (2023) suggested an increased abundance of AKK in the intestinal flora of the majority of patients with PD, with the abundance of other probiotics, such as Lactobacillus and Bifidobacterium, also changing accordingly. Thus, we find that an increase in AKK abundance may reduce the production of endotoxin and thus indirectly modulate neurotransmitter metabolism. However, it is unknown whether AKK synergizes with the other two probiotics to affect neurotransmitter metabolism and, consequently, Parkinson’s disease, which is an important area for further investigation. Additionally, exploring whether AKK competes with other components of the intestinal flora to promote neurotransmitter synthesis is worthwhile.

### Effects of *Akkermansia muciniphila* on short-chain fatty acids

SCFAs, including acetate, propionate, and butyrate, are the primary metabolic byproducts of gut microbiota fermentation and play pivotal roles in maintaining intestinal homeostasis, immune regulation, metabolic function, cardiovascular health, and neurobehavioural modulation (Dalile et al., 2019). These microbial metabolites communicate with the CNS through intestinal epithelial interactions, influencing neurological function (Ahmed et al., 2022). The gut microbiota produces SCFAs, primarily acetate, propionate, and butyrate, through dietary fibre fermentation. These SCFAs activate G protein-coupled receptor 41 and G protein-coupled receptor 43 on immune cells, thereby suppressing pro-inflammatory cytokine release and mitigating excessive immune responses (Maslowski et al., 2009). Among them, acetate and propionate share overlapping functions in immunomodulation, metabolic regulation, and neuroinflammation control (Maslowski et al., 2009). Both modulate neuroinflammation via vagal nerve signaling (Zheng et al., 2021; Zhang et al., 2023) and exert neuroprotective effects on sepsis-associated cognitive impairment through GPR43-mediated pathways that reduce immune cell overactivation and neuronal apoptosis (Liao et al., 2022). Moreover, acetate has therapeutic potential for autism spectrum disorders by altering the transcription of neuroplasticity-related genes in the prefrontal cortex (Osman et al., 2023), whereas propionate has been reported to exert cardioprotective effects via immune regulation in hypertension models (Bartolomaeus et al., 2019). In contrast, butyrate plays a distinct role in enhancing blood–brain barrier integrity (Zhang et al., 2023). Butyrate has emerged as a particularly remarkable neuroactive SCFA, with demonstrated efficacy in alleviating alcohol-induced neuroinflammation by shifting microglial polarization from the M1 to M2 phenotype and enhancing intestinal barrier function (Wei et al., 2023). Furthermore, butyrate regulates key neurotransmitters, including 5-HT, GABA, and DA, while also exerting epigenetic effects through histone deacetylase inhibition to promote neuroplasticity and cognitive function (Wang et al., 2020b; Kumar et al., 2021; Garvey, 2023).

Acetate appears to have multifaceted biological functions, including immunomodulation, metabolic regulation, and neurobehavioral effects through gut–brain axis communication (Xi et al., 2021). Within this broader analytical framework, acetate modulates neuroinflammation via vagal pathways and seems to generally protect cognitive function (Proano et al., 2023). Given the complexity of these theoretical relationships, acetate supplementation has therapeutic potential for autism spectrum disorders by ostensibly inducing transcriptional changes in the prefrontal cortex that, in the majority of cases, affect synaptic signaling and neuroplasticity-related genes (Dalile et al., 2019). Propionate, another crucial SCFA, has cardioprotective effects by attenuating hypertension-induced cardiac remodeling and vascular dysfunction through immune modulation (Ahmed et al., 2022). Butyrate has emerged as a particularly considerable neuroactive SCFA, with evidence that indicates its efficacy in alleviating alcohol-induced neuroinflammation by shifting microglial polarization from the M1 to M2 phenotype and enhancing intestinal barrier function (Zhang et al., 2023). Moreover, butyrate appears to regulate key neurotransmitters, including 5-HT, GABA, and DA, while seemingly exerting epigenetic effects through histone deacetylase inhibition that may promote neuroplasticity and cognitive function (Maslowski et al., 2009; Erny et al., 2021; Liao et al., 2022).

### *Akkermansia muciniphila* acts through the brain‒gut axis, thereby reducing the production of inflammatory factors

An increased abundance of AKK reduces the levels of inflammatory factors in several ways (Zhang et al., 2024c). AKK regulates the intestinal flora and decreases the levels of pro-inflammatory factors, which in turn reduces the inflammatory response in the nervous system (Silva et al., 2020; de la Rubia Ortí et al., 2023). One study has shown that live AKK exerts anti-inflammatory effects, reducing cognitive dysfunction in individuals with diabetes by decreasing pro-inflammatory factors and increasing the secretion of anti-inflammatory factors to mitigate damage to neuronal cells (Du et al., 2024). Experiments using ELISA validation of serum from mice treated with AKK revealed a downregulation of IL-6, TNF-α, and IL-1β in plasma, with IL-6 showing the most considerable reduction (Du et al., 2024). Additionally, inflammation-related pathways such as the NOD-like receptor, TNF, and mTOR signaling pathways were downregulated in the hippocampal tissues of mice, and IL-6 levels were also reduced (Zhu et al., 2023a). Another study isolated AKK from patients with multiple sclerosis and colonized it in mice to induce autoimmune encephalomyelitis (Cox et al., 2021). AKK was associated with the proportion of γδ T cells in the intestinal mucosa of RORγT^+^ mice, ultimately reducing IL-17 levels by decreasing its production (Cox et al., 2021).

AKK reduces the release of pro-inflammatory signals by modulating microglial activity and inhibiting microglial overactivation through its anti-inflammatory effects, which help attenuate neurological disorders (Cox et al., 2021). A study revealed that coculturing cells with AKK metabolites reduces serum levels of inflammatory factors and alleviates microglia-induced inflammation in the nervous system (Zou et al., 2023). Furthermore, Zhai et al. (2019b) cocultured the supernatants of two AKK strains with human HT-29 cells and found that the supernatant of strain ATCC or 139 significantly reduced IL-8 production in HT-29 cells. These findings suggest that both strains secrete substances that inhibit IL-8 production, indicating that both strains have anti-inflammatory properties *in vitro*. In another article, cells were cocultured with extracts from the fermentation of chicory and AKK, and the metabolites in the extracts were analyzed using nontargeted metabolomics (Chervet et al., 2025). The results showed that coculturing increased adiponectin secretion, reduced macrophage M1 polarization, and decreased IFNγ and IL-6 secretion. Additionally, AKK metabolites, such as SCFAs, seem to generally affect levels of anti-inflammatory neurotransmitters, such as 5-HT, thereby alleviating neurological issues caused by neurotransmitter imbalances (Chervet et al., 2025). Within these evolving conceptual parameters, an increased abundance of AKK tends to be associated with elevated levels of 5-HT in the gut and brain, along with decreased expression of pro-inflammatory factors (Chervet et al., 2025). This relationship provides evidence that may support an improvement in symptoms such as depression, given the complexity of these theoretical relationships (Guo et al., 2024). Moreover, some AKK metabolites activate the vagus nerve and transmit anti-inflammatory signals to the CNS via the vagal pathway, thereby attenuating associated neurological behaviors. These findings indicate that coculturing cells with AKK products effectively reduces levels of inflammatory factors (Guo et al., 2024).

Recent experiments using a mouse model of liver injury have indicated that the level of 5-HT in the vagus nerve is increased in AKK-treated mice, suggesting that AKK reduces the release of inflammatory factors by modulating the vagus nerve, ultimately decreasing the occurrence of neuroinflammation (Kang et al., 2024a). In another experiment, Caco-2 cells were treated with AKK metabolites and then cocultured with HMC3 cells to construct an *in vitro* brain–gut axis. The results showed that AKK metabolites reduced levels of the cytokines IL-6 and IL-17A, suggesting that AKK can alleviate neuroinflammation caused by microglia (Zou et al., 2023). In a mouse model of sleep deprivation, the levels of AKK in the intestines decreased, along with levels of acetate and butyrate among SCFAs (Li et al., 2023). An increase in SCFA levels altered Iba1-positive staining in the hippocampus, reducing the number of positive points in microglia, and an assessment of the behavioural performance of these mice indicated that cognitive dysfunction was alleviated in mice pretreated with SCFAs (Li et al., 2023). All of these pathways contribute to reducing levels of inflammatory factors; however, several questions remain to be addressed. First, while an increase in AKK abundance is associated with reduced levels of inflammatory factors such as IL-6 and TNF-α, there has been limited discussion on whether AKK modulates specific immunoregulatory pathways, such as the Treg/Th17 balance and NF-κB signaling. Second, do different doses of AKK influence the levels of inflammatory factors? Third, do various health states (with or without underlying diseases) influence inflammatory factors in patients? These issues warrant further exploration in future experiments.

## Role of *Akkermansia Muciniphila* in Neurologically Related Diseases

### Alzheimer’s disease

**Figure 4:**
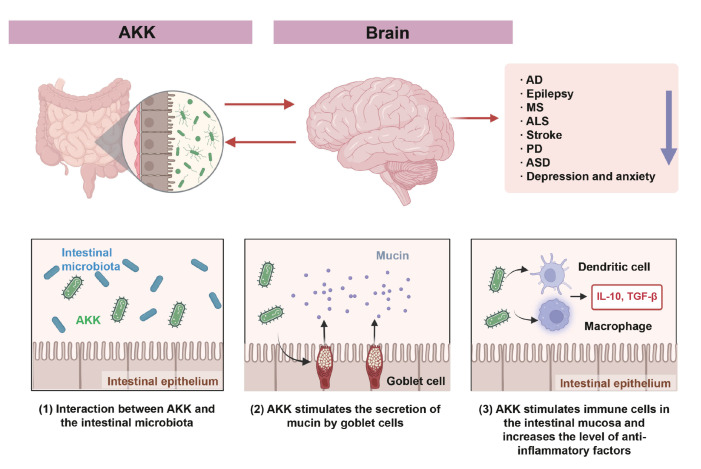
AKK affects neurological disorders by regulating the brain‒gut axis. First, AKK interacts with other components of the gut microbiota by stimulating goblet cells to secrete mucin, maintaining the thickness of the intestinal barrier within a normal range, and stimulating immune cells in the intestinal mucosa, such as macrophages and dendritic cells, to increase the levels of anti-inflammatory factors. Neurological disorders such as AD, epilepsy, MS, ALS, stroke, PD, ASD, depression, and anxiety are ultimately alleviated through the brain‒gut axis. Created with BioRender.com. AD: Alzheimer’s disease; AKK: *Akkermansia muciniphila*; ALS: amyotrophic lateral sclerosis; ASD: autism spectrum disorder; MS: multiple sclerosis; PD: Parkinson’s disease.

AD is a neurodegenerative disorder that primarily affects the elderly population, commonly manifesting as abnormal speech, memory loss, and cognitive impairment (Scheltens et al., 2021). In patients with AD, substantial amounts of Aβtend to accumulate in the brain, forming amyloid plaques that disrupt intercellular signaling between neurons, leading to neuronal dysfunction (Scheltens et al., 2021). Notably, neuronal loss, neuroinflammation, and neurofibrillary tangles can greatly exacerbate what may be characterized as neuronal damage and death (Khan et al., 2020; Ma et al., 2022). Within this broader analytical framework, the most critical factors in the pathogenesis of AD include Aβ accumulation, oxidative stress, and mitochondrial dysfunction. In light of these methodological considerations, a recent study has indicated that changes in the gut microbiota are associated with the onset and progression of AD, suggesting that dysbiosis of the gut microbiota may serve as an early preclinical marker of the disease (Ferreiro et al., 2023). Additionally, the concept of the brain-gut axis has been increasingly introduced, proposing that disorders in gut flora may contribute to the development of AD, and that supplementation with gut probiotics could be a promising therapeutic strategy. A study examining a ketogenic and low-fat diet in older adults at risk of AD suggests that a ketogenic diet may improve cognitive function and gut health in individuals with AD by modulating the gut microbiota, particularly by increasing the abundance of AKK (Dilmore et al., 2023). The relationship between dietary interventions and microbial composition is especially noteworthy, given the multifaceted nature of this evidence and the theoretically significant connections between nutrition and neurological outcomes (Dilmore et al., 2023; **Figure 4**). Furthermore, a study investigating AKK in the treatment of various neuropsychiatric disorders revealed that AKK modulates the gut–brain axis, thereby reducing neuroinflammation, promoting the production of metabolites such as SCFAs, and ultimately improving cognitive dysfunction associated with AD (Xu et al., 2023). Additionally, a systematic evaluation and meta-analysis utilizing 16S rRNA sequencing to observe changes in the gut flora of AD patients found that the abundance of AKK was higher in these patients, suggesting that AKK has great potential as a probiotic for the treatment of AD (Li et al., 2024a, b).

### Epilepsy

Epilepsy is a neurological disorder characterized by abnormal neuronal discharges in the brain. It is recurrent and has a complex pathogenesis involving metabolism, genetics, and immunity (Falco-Walter, 2020). The main pathological feature of epilepsy is a reduction in inhibitory neurotransmitters and an increase in excitatory neurotransmitters in the brain, leading to neuronal overexcitation and ultimately resulting in seizures (Sears and Hewett, 2021). Additionally, inflammatory responses and oxidative stress in the brain exacerbate seizure frequency by damaging neurons (Parsons et al., 2022). The gut microbiota also plays an important role in the treatment of epilepsy. One study has shown that ketogenic diets lead to considerable changes in intestinal flora, particularly in the ratio of AKK to *Parabacteroides*, both of which colonize the gut and contribute to protection against epilepsy (Olson et al., 2018). The increased abundance of AKK leads to a reduction in the production of γ-glutamyl-amino acids, ultimately resulting in an elevated GABA/glutamate ratio, which exerts an antiepileptic effect (Cheng and Xie, 2021). Gong et al. (2021) investigated children with epilepsy who had developed drug resistance and found that these children exhibited dysbiosis in their gut microbiota, as well as a reduced abundance of *Akkermansia*. Following treatment interventions, the changes in gut microbiota levels were reversed, and the levels of SCFAs increased remarkably. Additionally, another study suggested that bacterial cultures of AKK generally inhibit the expression of drug resistance genes (Ilhan et al., 2022). Within this broader analytical framework, increased expression of drug resistance genes can lead to resistance to antiepileptic drugs in people with epilepsy. AKK appears to inhibit the expression of these drug resistance genes, potentially slowing drug resistance and contributing to epilepsy treatment approaches. Given the complexity of these theoretical relationships, these findings are significant for managing therapeutic efficacy in patients receiving long-term antiepileptic treatment. Although few reports focus specifically on AKK and its antiepileptic effects, the findings above indirectly suggest that AKK plays a role in antiepileptic treatment. This warrants further exploration of the relationship between AKK and epilepsy in future studies.

### Multiple sclerosis

MS is a common autoimmune disease that affects the CNS, often leading to impaired neurological function (Olek 2021). The main pathological feature is the attack of the myelin sheath of neurons by a variety of immune cells, including T cells, B cells, and macrophages (Woo et al., 2024), which play important roles in the destruction of the myelin sheath, the inflammatory response, and nerve damage (Rodríguez Murúa et al., 2022). A recent study using a mouse model of experimental autoimmune encephalomyelitis demonstrated that AKK in the gut microbiota plays a key role in MS and that the abundance of AKK in the faeces serves as a predictor of the severity of experimental autoimmune encephalomyelitis (Steimle et al., 2024). The experiment also revealed that the immunoglobulin A encapsulation index of a specific colony can predict the progression of the disease; therefore, the abundance of AKK may become an important factor in predicting MS severity in the future (Steimle et al., 2024). Another study provided insights into how gut microbes in patients with MS regulate T-cell function and exacerbate MS symptoms in a mouse model and revealed that AKK abundance was considerably increased in patients with MS, increasing immune-related symptoms of MS through the activation of proinflammatory responses in peripheral blood mononuclear cells (Cekanaviciute et al., 2017). An experiment in which microRNA-30d (miR-30d) was enriched from the stool samples of patients with MS suggested that miR-30d generally promoted the proliferation of AKK, resulting in a potential alleviation of the corresponding symptoms through an increase in the number of T cells (Liu et al., 2019). The relationship between miR-30d and symptom reduction is particularly noteworthy in this context (Liu et al., 2019). However, within this broader analytical framework, the specific mechanism of action of miR-30d in the intestinal tract remains largely unclear, and how to presumably regulate miR-30d with precision warrants further interpretive consideration in future research endeavours, given the multifaceted nature of this evidence (Liu et al., 2019).

### Amyotrophic lateral sclerosis

Amyotrophic lateral sclerosis (ALS), also known as Lou Gehrig’s disease, is a progressive neurodegenerative disorder characterized by the gradual damage to motor neurons, leading to muscle weakness, atrophy, and potentially respiratory failure (Grad et al., 2017; Feldman et al., 2022). ALS has a remarkably complex etiology, and while the exact mechanisms remain somewhat unclear, it is generally associated with both genetic and environmental factors within the broader context of multifactorial disease development (Feldman et al., 2022). Importantly, ALS involves dysregulated immune responses, neuroinflammatory processes, oxidative stress, and mitochondrial dysfunction (Feldman et al., 2022). For instance, evidence regarding the immune mechanisms of ALS suggests that once astrocytes and microglia become activated, they release substantial amounts of inflammatory factors. This not only leads to neuroinflammatory reactions but also results in damage to motor neurons, based on the complex relationships between neuroinflammation and neurodegeneration (Giacomelli et al., 2022). Recent research has indicated a considerable link between gut flora and ALS (Blacher et al., 2019). Specifically, it has been shown that approximately 11 species of gut commensal bacteria are associated with the disease severity in many individuals with ALS (Blacher et al., 2019). Within this broader context, AKK appears to alleviate ALS symptoms, while some other species may exacerbate them (Blacher et al., 2019). AKK tends to ameliorate symptoms in mice by increasing nicotinamide levels in the CNS, which seems to help regulate neuronal metabolism (Blacher et al., 2019). Another study found that AKK not only affects nicotinamide levels in a mouse model to improve motor symptoms but also results in lower levels of nicotinamide in the blood and cerebrospinal fluid of patients with ALS in a preliminary human study, providing a theoretical basis for future large-scale clinical studies (Gotkine et al., 2020). One study revealed dysbiosis of the gut microbiota in transgenic mice prone to ALS (Casani-Cubel et al., 2021). AKK is positively correlated with *Verrucomicrobia* levels, and an increase in *Verrucomicrobia* abundance can reduce inflammatory factor levels in serum and animal tissues, benefiting patients with ALS (Casani-Cubel et al., 2021). Additionally, Pribac et al. (2024) have reported that the abundance of AKK was reduced in individuals with ALS and that increasing these bacteria alleviated the severity of ALS in mice, possibly through nicotinamide production. Although relevant experiments have shown that AKK and its product, nicotinamide, improve symptoms in patients with ALS, the influence of other confounding variables, such as diet and lifestyle, cannot be ruled out in preliminary human studies. Future research will involve a large-scale analysis of patients to eliminate the influence of these factors.

### Stroke

Stroke, an acute neurological disorder caused by the rupture of a cerebral blood vessel or obstruction of blood flow to the brain, can generally be divided into two types: ischemic stroke and hemorrhagic stroke (Murala et al., 2022; Feigin et al., 2025). Evidence suggests that ischemic strokes result from an obstruction of blood vessels in the brain, which leads to ischaemia and hypoxia of the brain tissue, whereas hemorrhagic strokes stem from a rupture of blood vessels in the brain, leading to intracerebral hemorrhage (Maida et al., 2024). Patients with stroke typically exhibit symptoms such as hemiparesis, speech impairment, cognitive impairment, and unsteady gait, which may lead to death in the majority of patients with severe cases (Zhao et al., 2022). These conclusions indicate that AKK therapy has a positive effect on the prognosis of patients with stroke. Within this broader analytical framework, a study using 16S rRNA sequencing to analyze changes in the gut microbiota of a mouse model of ischemic stroke indicates that AKK supplementation is capable of partially restoring the balance of the intestinal flora (Wang et al., 2025b). Notably, this restoration appears to lead to a reduction in the area of injury, a substantial decrease in the number of neuronal and apoptotic cells in the necrotic area, a restoration of locomotor ability, and a decrease in the overactivation of microglia (Wang et al., 2025b). Research suggests that live AKK may mitigate brain damage caused by stroke by reducing the cerebral infarct area and enhancing the integrity of the intestinal barrier, potentially alleviating the occurrence of stroke in many cases (Wang et al., 2025b). Within this context, the richness of indoleacetic acid in the AKK supernatant appears to play an important role, indicating its ability to activate the AhR and Nrf2 pathways (Wang et al., 2025b). Given the complexity of these theoretical relationships, this activation tends to upregulate the expression of the SLC7A11 and GPX4 proteins, subsequently inhibiting ferroptosis, which suggests a substantial reduction in the severity of stroke (Wang et al., 2025b). However, how these pathways interact under various physiological conditions through a complex regulatory mechanism remains unclear. Considering the nuanced nature of these findings, indoleacetic acid may function through multiple complementary mechanisms (Rahman et al., 2024). Whether these effects can be reproduced across different models or if they represent a context-specific phenomenon warrants further consideration (Rahman et al., 2024). In light of these methodological considerations, this analysis suggests that AKK supplementation could be a novel therapeutic approach for post-stroke recovery paradigms. Although AKK appears to have beneficial effects, its abundance seems to decrease in stroke rats after certain synbiotic interventions, providing evidence that may support the notion that AKK is not always beneficial and may be interrelated with a certain balance (Rahman et al., 2024).

Additionally, Li et al. (2024b) have shown that AKK expression increases considerably after ischemic stroke and that supplementing AKK in advance can reduce stroke-induced damage, ameliorate neuroinflammation, and improve the prognosis of mice. These findings suggest that AKK can play a protective role in stroke by regulating neuroinflammation. Another experiment also confirmed the relationship between the gut flora and stroke; high-throughput genome sequencing was used to analyze species changes in the gut microbiota, and the abundance of beneficial bacteria, such as AKK, was considerably reduced after stroke (Hammond et al., 2022). Within this broader analytical framework, related experiments have shown through a correlation analysis that AKK is negatively correlated with the majority of bacterial genera (Stanley et al., 2018). Importantly, in an experiment conducted 24 hours after stroke in mice, AKK was found to be predominantly positively correlated with certain *Bacteroides* and *Parabacteroides genera* but apparently negatively correlated with *Staphylococcus* and *Streptococcus genera* (Stanley et al., 2018). Therefore, this pattern suggests that AKK plays a positive role after a stroke by presumably preventing pathogens from migrating to epithelial cells, thereby reducing lung infections and dissemination in mice following a stroke. However, given the complexity of these theoretical relationships, the connection between AKK and stroke remains unclear, as conclusions regarding its abundance in human faecal samples and animal models poststroke tend to vary across different investigations (Ma et al., 2025a). While some data suggest that AKK levels typically increase after stroke, a report indicates a decrease, highlighting the need for further studies to clarify this inconsistency (Ma et al., 2025a). Research suggests that, AKK levels in the gut generally decrease during the treatment of cerebral ischemia in rats with multistrain probiotics, although whether this reduction results from changes in AKK abundance itself or from the combined effects of probiotic strains remains uncertain (Ma et al., 2025a). Within this broader analytical framework of microbial interactions, this relationship warrants further investigation due to the multifaceted nature of the current evidence. All of these studies suggest that AKK plays a role in the recovery of the stroke course and lay the groundwork for subsequent treatment of this disease using the gut flora.

### Parkinson’s disease

PD is a neurodegenerative disorder characterized by tremor, rigidity, bradykinesia, and postural instability. It may also present with symptoms including cognitive dysfunction, mood swings, and depression (Kalia and Lang, 2015). The prevalence of PD in the global population is approximately 1 in 1000, but this rate increases to about 1% in patients over 60 years of age (Tysnes and Storstein, 2017). Potential mechanisms underlying PD include damage to and loss of DA neurons in the substantia nigra region (Zhu et al., 2022), neuroinflammatory and immune responses, oxidative stress, and changes in the brain-gut axis (Pajares et al., 2020). AKK, a probiotic typically found in the gut, appears to protect against damage by restoring the stability of the gut barrier, thereby reducing neuroinflammation and presumably minimizing the entry of toxins into the brain (Effendi et al., 2022). A recent study investigating intestinal differences between patients with PD and healthy controls in three different geographic regions suggested a substantial correlation between PD and dysbiosis of the intestinal flora in a predominantly Western patient population (Proano et al., 2023). Notably, within this broader analytical framework, AKK specifically seems to be strongly associated with the disease in the majority of patients from all three regions (Proano et al., 2023). Given the complexity of these theoretical relationships, the evidence reveals a potentially important connection that, considering the nuanced nature of these findings and methodological considerations, warrants further investigation (Proano et al., 2023). Another experiment examining the differences between two subtypes of PD (body-preferred and brain-preferred) in relation to gut flora dysbiosis suggested that AKK counts were significantly increased in patients with body-preferred PD, whereas gut flora appeared to be less altered in patients with brain-preferred PD compared to healthy controls (Nishiwaki et al., 2024). After adjusting for confounding factors in data from Japan and the United States, a meta-analysis revealed a significant increase in the number of AKK in patients with PD (Nishiwaki et al., 2024). Importantly, a meta-analysis of other gene sequencing results also indicated an increase in AKK counts among patients with PD from different countries (Nishiwaki et al., 2024). We speculate that this result may be due to AKK compensating for the reduced levels of SCFAs in patients with PD by increasing its own abundance in this environment. Patients with PD are typically classified into motor-onset and brain-onset types (Horsager et al., 2022). Evidence suggests that the abundance of AKK in the gut of patients with motor-onset PD increases substantially, while this increase is less pronounced in patients with brain-onset PD. AKK levels in patients with both types of PD may be closely related to the pathogenesis of motor-dominant PD (Park et al., 2024). These evolving conceptual parameters indicate that patients with motor-dominant PD often exhibit isolated rapid eye movement sleep behavior disorder, and the increase in AKK abundance in the intestines of patients with idiopathic rapid eye movement sleep behavior disorder also seems to suggest dysbiosis in the early stages of motor-dominant PD (Park et al., 2024).

Recent research suggests that the probiotic AKK may improve motor dysfunction in a mouse model of PD by restoring dopaminergic neurons in the brain, promoting microglial activation, and reducing levels of inflammatory factors and colon damage (Wang et al., 2025c). In a mouse model of PD, AKK ingestion was associated with reduced bradykinesia and appeared to inhibit dopaminergic neuron loss in the substantia nigra, although this effect was not consistent across all cases. The treatment largely restored tyrosine hydroxylase expression in the same region (Wang et al., 2025c). Notably, AKK also attenuated neuroinflammatory responses in the striatum and hippocampus, highlighting its multifaceted therapeutic potential in the complex neuroimmune landscape of PD. Another study analyzed fecal and nasal samples from 76 patients with PD, 21 patients with rapid eye movement sleep behavior disorder, and 78 healthy controls (Heintz-Buschart et al., 2018). The results showed that the abundance of AKK in the fecal flora of patients with PD was increased (Heintz-Buschart et al., 2018). We speculate that the increase in AKK abundance may lead to mucus layer depletion, thereby increasing intestinal permeability and susceptibility to pathogens, which may promote α-synuclein deposition. One investigation suggests that MPTP typically reduces the abundance of AKK because AKK can produce secretory immunoglobulin A and antimicrobial peptides through immune rejection reactions, thereby reducing pathogen-induced damage to the intestine and alleviating PD symptoms (Fang et al., 2020). A cohort study revealed that AKK seems to accumulate in individuals with neurodegenerative diseases, possibly increasing intestinal inflammation and permeability through mucin degradation. This suggests that systemic inflammation may contribute to the progression of neuropathology (Zapała et al., 2021). Within this broader analytical framework, and considering the nuanced nature of this finding, the investigation appears to suggest that AKK may be associated with PD, but this association might also stem from increased intestinal permeability, ostensibly resulting from increased mucus degradation by AKK (Zapała et al., 2021). Moreover, given the multifaceted nature of this evidence, AKK seems to substantially modulate SCFA levels in most mice, a phenomenon that is closely associated with its apparent effects on neuroinflammation and neurological responses (Qiao et al., 2024). Therefore, the data suggest that AKK tends to attenuate neuroinflammation and nervous system dysfunction (Qiao et al., 2024). These findings indicate that AKK plays a potentially promising role in alleviating PD symptoms.

### Autism spectrum disorder

Autism spectrum disorder (ASD) is a neurodevelopmental disorder characterized by impairments in social interaction and communication, stereotyped behaviors, and more restricted interests (Lord et al., 2018; Jiang et al., 2022). In recent years, as research has continued, increasing evidence has shown that the development of ASD is linked to a variety of factors, including neurotransmitters in the brain, gene mutations, and environmental influences (Hirota and King, 2023). From a genetic perspective, the occurrence of ASD is often closely related to mutations or deletions of specific genes (Wang et al., 2023a). One study confirmed the effect of fecal microbiota transplantation on autism-like behaviors in knockout mice (Goo et al., 2020). Social avoidance behaviors and memory deficits in these mice were obviously reduced by increasing the abundance of specific components of the intestinal flora, particularly AKK, through fecal microbiota transplantation. This process promoted the expression of MUC2 in the intestinal mucus layer, contributed to the restoration of the intestinal barrier, and thus alleviated the clinical condition (Goo et al., 2020). Additionally, a study exploring whether a ketogenic diet could modulate changes in gut flora, particularly AKK, and consequently alter the behavior of mice with ASD suggests a notable relationship (Newell et al., 2016). Within this broader analytical framework, a ketogenic diet substantially reduces the total amount of gut microbes in mice. Importantly, a ketogenic diet appears to reverse the abnormally high levels of AKK in these mice and restore the normal distribution of gut flora, which, given the complexity of these theoretical relationships, seems to ameliorate the symptoms of ASD in the majority of subjects examined (Newell et al., 2016). Furthermore, a study compared 48 autistic children in China with a control group and reported that the abundance of *Verrucomicrobia* was obviously lower in the autistic group, with AKK levels also lower than those in the control group (Heintz-Buschart et al., 2018). In addition, a survey analyzed the relationship between intestinal flora and neurobehavioral performance in children with ASD through microbiome statistical modeling (Jendraszak et al., 2021). The results showed that the abundance of AKK in children with ASD differed remarkably from that in control children and was significantly correlated with social skills and repetitive behaviors, indicating that AKK may play a key role in the brain-gut axis mechanism of ASD. Fecal samples were collected from children with ASD, and the abundance of several specific gut bacteria was measured; results suggested a substantially lower abundance of AKK in children with ASD compared with neurotypical children (Wang et al., 2011). This pattern suggests that AKK may promote intestinal health. Within this broader analytical framework, these findings support an association between low AKK abundance and common intestinal symptoms, such as constipation and diarrhea, prevalent among many children with ASD (Wang et al., 2011). Given the complexity of these theoretical relationships, further studies of the potential mechanistic pathways involved are warranted. Moreover, a meta-analysis of studies on the intestinal flora of multiple ASD and control groups revealed that the abundance of AKK was substantially reduced in patients with ASD, suggesting that AKK may be related to impaired integrity of the intestinal mucus layer (Xu et al., 2019a). Agarwala et al. (2021) found that the abundance of AKK in the feces of children with autism was remarkably reduced and negatively correlated with indicators of intestinal inflammation. This result further suggests that AKK deficiency may be involved in the pathogenesis of ASD by compromising the intestinal barrier and exacerbating inflammation. Although these experiments provide evidence for the association between AKK abundance and ASD, the exploration of the specific mechanisms is insufficient, and technologies such as transcriptomics, proteomics, and metabolomics need to be implemented to study the relationship between gut microbiota and autism in detail. Additionally, while AKK supplementation can lead to short-term improvements, questions remain regarding its long-term efficacy and potential side effects, warranting further in-depth research.

### Depression and anxiety

Depression and anxiety are common mental disorders, and in recent years, strong links have been identified between gut flora and these conditions (Bauer and MacNamara, 2021). Depression is a prevalent affective disorder characterized by sadness, loss of interest, fatigue, and changes in sleep and appetite (Figee et al., 2022). The pathological mechanism of depression remains unclear, but studies have shown that it is related to neurotransmitter dysregulation, neuroinflammation, and the brain–gut axis (Penninx et al., 2021; Sarno et al., 2021; Chang et al., 2022). Research has suggested that 5-HT levels tend to be reduced in patients with depression (Sarno et al., 2021). Importantly, 5-HT is produced from its precursor, tryptophan. When inflammation occurs, tryptophan is metabolized through the kynurenine pathway, which may contribute to reduced 5-HT levels. In this context, gut microbiota dysbiosis in patients with depression can be linked to BDNF through the brain–gut axis (Chang et al., 2022). Multiomics analyses suggest that specific gut microbiota and metabolites may serve as biomarkers for depression. In contrast, anxiety is a mood disorder characterized by excessive worry, fear, and tension, which can overlap with symptoms of depression (Penninx et al., 2021). The pathogenesis of anxiety is complex, typically involving genetics, environmental factors, abnormalities in brain structure, and the cerebral–intestinal axis (Penninx et al., 2021). Given the multifaceted nature of this evidence, macrogenomic sequencing of rat feces after sleep deprivation has shown that sleep-deprived rats exhibit obvious anxiety-like behaviors and an increased abundance of certain gut microbiota species, along with a dramatic decrease in AKK levels, elevated lipopolysaccharide (LPS) concentrations, and reduced tryptophan levels (Zhang et al., 2024b). After 14 days of AKK administration, serum LPS levels decreased, and anxiety-like behaviors improved slightly, suggesting that AKK might alleviate anxiety symptoms by modulating the immune response. This highlights a connection between microbiota composition and behavioral manifestations within this broader analytical framework (Zhang et al., 2024b). Another meta-analysis reviewed 15 animal studies and concluded that AKK and its products can reduce symptoms of anxiety and depression while improving memory (Khalili et al., 2025). Additionally, a study reported that in an alcohol-induced depression mouse model, AKK considerably improved depression-like behaviors by strengthening the intestinal barrier and reducing LPS levels and neuroinflammation (Guo et al., 2022). The evidence suggests that AKK ameliorates depression-like behavior in mice through modulation of the gut microbiota and metabolites. Notably, AKK appears to modulate the nervous system by restoring neurotransmitter homeostasis, adjusting cortisol and DA levels, and increasing the expression of BDNF, potentially alleviating depressive symptoms (Ding et al., 2021). Although these findings provide evidence that AKK modifies depressive and anxiety behaviors, it is important to rationally optimize the experiments regarding sample size, explore the underlying mechanisms, and analyze different subtypes of the diseases. Based on these methodological considerations, AKK may be a promising probiotic for treating degenerative diseases in the future.

Based on the previously mentioned effects of AKK on the brain–gut axis, **Additional Table 1** complements and summarizes the areas of research, study targets, and clinical interventions involving AKK in neurological disorders, classifying representative disorders and related literature.

**Additional Table 1:** Summary of AKK and clinically relevant neurological disorders

Disease	Research subject	Type of research	Clinical intervention	Study conclusion	Reference
PD	MPTP-induced mouse model of PD	Animal experiments	Continuous 21-d gavage supplementation of mice with AKK	AKK attenuates neuroinflammation and promotes neurogenesis in the hippocampus, and improves motor function in a mouse model of PD.	Qiao et al., 2024
Type 2 diabetes and AD	A zebrafish model of type 2 diabetes and AD induced by a high-glucose diet and AlCl3 solution	Animal experiments	Daily gavage supplementation of Zebrafish with pasteurized AKK for 60 d	Pasteurized AKK considerably improved blood glucose levels and memory function and reduced anxiety, aggressive behaviors and AD-like symptoms.	Qu et al., 2023a
Epilepsy	Routine rearing, aseptic rearing and antibiotic-treated mouse models	Animal experiments	Mice fed a ketogenic diet and treated with antibiotics received partial microbial transplants of AKK and *Parabacteroides*	Supplementation with AKK and *Parabacteroides* restored anticonvulsant ability, increased the hippocampal GABA/glutamate ratio and attenuated the seizure frequency in epileptic mice.	Olson et al., 2018
ASD and other neurodevelopmental disorders	1197 infants diagnosed with neurodevelopmental disorders	Analysis ofthe gut microbiome and serum metabolites	No direct intervention was administered and the relationship between antibiotic use, maternal and infant infections and the flora was analyzed.	AKK was more abundant in healthy individuals, and a reduction in AKK abundance may be associated with an elevated risk of developing autism.	Ahrens et al., 2024
Clinical depression	A male mouse model of depression induced by chronic restraint stress	Animal experiments	Mice were treated with AKK for 3 wk and behavioral performance and neurotransmitter levels were measured	AKK improved depression-like behaviors and restored BDNF, dopamine and corticosterone levels.	Ding et al., 2021
ALS	Sod1-Tg transgenic mouse model and human patients with early-stage ALS	Animal experiments, observational studies in patients with ALS	Assessment of motor function and survival time in mice treated with broad-spectrum antibiotics and supplemented with AKK.	AKK improved locomotion and survival time in mice and reduced ALS symptoms by increasing nicotinamide levels in the nervous system.	Blacher et al., 2019
Anxiety, depression	Antibiotic-treated mouse model ofanxiety and depression-like behaviors	Animal experiments	Behavioural and biochemical indices in mice supplemented with AKK and the Amuc 1100 protein	AKK and Amuc_1100 remarkably alleviated antibiotic-induced anxiety and depression-like behaviors.	Sun et al., 2023
AD	A rat model of D-galactose/AlCl3-induced AD	Animal experiments	Oral administration of AKK to assess its effects on cognitive function, inflammation and metabolism	AKK improved cognitive function and pathological symptoms in AD-like rats by improving the intestinal flora diversity and reducing inflammatory factor levels.	He et al., 2022b
Ischemic stroke	C57BL/6 mice and CX3CR1GFP/+ transgenic mice	Animal experiments	Mice were administered an AKK suspension of at 2 × 10^9^ CFUs per dose by gavage daily for 7 d prior to stroke surgery	AKK reduced the cerebral infarct size, neuronal degeneration and density of apoptotic cells, inhibited microglia activation, promoted the expression of anti-inflammatory factors, and improved behavioral performance after stroke.	Li et al., 2024b
MS	Autoimmune encephalomyelitis mice	Animal experiments	The effect of miR-30d on MS-like symptoms in mice was investigated by faecal transfer from patients with MS orally administered AKK	Faeces from patients with MS enhanced the effect of AKK via miR-30d to alleviate MS-like symptoms in mice.	Liu et al., 2019

AD: Alzheimer's disease; AKK: *Akkermansia muciniphila*; ALS: amyotrophic lateral sclerosis; ASD: autism spectrum disorder; BDNF: brain-derived neurotrophic factor; CFU: colony-forming unit; GABA: gamma-aminobutyric acid; AlCl3: aluminium chloride; MPTP: 1- methyl-4-phenyl-1:2:3:6-tetrahydropyridine; MS: multiple sclerosis; PD: Parkinson's disease; Sod1-Tg: superoxide dismutase 1 transgenic.

## Regulation of the Brain–Gut Axis and the Nervous System by Different Forms of *Akkermansia Muciniphila*

AKK appears to exist not only in the form of intact live bacteria but also in various alternative forms, including outer membrane proteins, extracellular vesicles, and pasteurized inactive bacteria (Raftar et al., 2022). The evidence suggests that these different forms of AKK regulate intestinal function, immune responses, and neurological signaling through distinct mechanisms, potentially affecting brain–gut axis function and neurological disorders within this broader analytical framework (Lei et al., 2023). Notably, these various forms of AKK seem to be clinically effective in the treatment of obesity (Liu et al., 2024), metabolic diseases (Xiao et al., 2024), and liver diseases (Wu et al., 2025). These findings have also paved the way for a meaningful exploration of the effects on neurological disorders. Given the complexity of these theoretical relationships, we explore the specific mechanisms of the different forms of AKK and their respective strengths and weaknesses, including their effects on the brain–gut axis, in the following sections to provide conceptual foundations for future clinical treatment of neurological disorders.

### Amuc_1100

Outer membrane proteins are proteins on the outer membrane of bacteria and appear to be more common in gram-negative bacteria than in gram-positive bacteria because the outer membrane seems to be one of their unique cellular structures (Rollauer et al., 2015). These proteins play important roles in bacterial physiological processes, including material exchange, information transfer, and interactions with host cells. The outer membrane proteins of AKK have been reported to be expressed in several different forms, namely, Amuc_1100, Amuc_1409, Amuc_1102, Amuc_2109, and Amuc_1434, each of which seems to perform a unique physiological role within this broader analytical framework (Meng et al., 2020; Wang et al., 2021; Xiang et al., 2021; Qian et al., 2022; Kang et al., 2024b). Comparatively, Amuc_1100 has received substantial attention from researchers because it apparently plays an important role in bacterial‒host interactions and tends to be closely and intimately associated with a wide range of clinical diseases, especially those that might be characterized as metabolic system–related diseases and immune system diseases (Plovier et al., 2017; Wang et al., 2020c). Amuc_1100, an outer membrane protein of AKK, has been shown to regulate metabolic pathways in adipocytes, ameliorate fat accumulation and metabolic function problems associated with obesity, and activate the AC3/PKA/HSL signalling pathway, which suggests its contribution to lipolysis and browning (Zheng et al., 2023b). Another study suggested that Amuc_1100 presumably binds to the TLR2 receptor of the immune system and typically activates the immune response in this host, an effect that not only seems to improve the function of the intestinal barrier but also potentially attenuates the chronic inflammatory response induced by obesity and diabetes, given the complexity of these theoretical relationships (Plovier et al., 2017; **Additional Table 2**).

**Additional Table 2:** Characteristics, advantages, and disadvantages of different forms of AKK in treating different diseases

Form	Characteristic	Related diseases	Dominance	Disadvantage	Reference
Live AKK bacteria	Colonize the intestinal tract and interact directly with the host's intestinal flora to maintain long-term microbial balance	Neurodegenerative diseases, chronic inflammation, metabolic disorders, intestinal immune response, atherosclerosis, liver fibrosis, and cancer.	Long-term colonization, stable regulation of the flora and intestinal barrier function.	Unstable activity, susceptible to environmental influences, requir suitable storage conditions.	Li et al., 2016; Ashrafian et al., 2021b; Keshavarz Azizi Raftar et al., 2021; Peña-Cearra et al., 2024; Zhu et al., 2024
Outer membrane proteins (Amuc_1100)	Present on the outer membrane of AKK bacteria, they bind to the TLR2 receptor, enhance the tight junctions of the intestinal epithelium, and regulate the body's immune response.	Depression, anxiety, cognitive disorders, inflammatory bowel disease, metabolic disorders, anxiety, lung adenocarcinoma.	Directly interact with intestinal epithelial cells, enhance intestinal barrier integrity and reduce permeability; regulate the brain-gut axis.	Dependent on the intestinal environment and require contact with host cells for the maximum effect; dependent on the TLR2 signaling pathway.	Wang et al., 2021a; Zhang et al., 2022a; Sun et al., 2023; Wade et al., 2023; Kang et al., 2024b; Xu et al., 2024; Zhang and Wang 2023
EVs	Contain proteins, RNA, lipids and other substances that regulate intestinal permeability and metabolism.	Postoperative cognitive dysfunction, prostate cancer, obesity, diabetes, and inflammatory bowel disease.	Easily absorbed orally, they act without direct contact with host cells.	Complicated preparation process.	Chelakkot et al., 2018; Ashrafian et al., 2019; Luo et al., 2021; Díez-Sainz et al., 2022; Yaghoubfar et al., 2023; Gao etal., 2024
Pasteurized AKK	AKK retains some of its activity after pasteurization, improving metabolic and immune functions and strengthening the intestinal barrier.	AD, irritable bowel syndrome, colitis, and metabolic diseases.	It is easily absorbed after oral administration and does not pose a risk of infection.	Not as effective as live bacteria.	Wang et al., 2020; Zou and Chen 2020; Druart et al., 2021; Qu et al., 2023b; Meynier et al., 2024;
AKK in combination with other probiotics	Regulate the intestinal flora, enhance intestinal barrier function, and reduce inflammation and lipid accumulation.	NAFLD, metabolic diseases, and intestinal inflammation.	The combination treatment is more potent and effective than monotherapy.	The ratio of AKK to *Bifidobacteria* needs to be optimized in advance.	Nian et al., 2023

AD: Alzheimer's disease; AKK: *Akkermansia muciniphila;* EVs: extracellular vesicles; NAFLD: nonalcoholic fatty liver disease; RNA: ribonucleic acid; TLR2: Toll-like receptor 2.

The evidence suggests that Amuc_1100 attenuates intestinal mucositis in mice by reducing the levels of inflammatory factors, increasing the expression of tight junction proteins, and largely restoring the integrity of the intestinal tract, which provides evidence supporting the therapeutic potential of this outer membrane protein (Chen et al., 2022). These findings suggest that the outer membrane protein Amuc_1100 functions in a variety of diseases. In addition to its role in metabolic diseases, studies have shown that Amuc_1100 can also regulate diseases related to the nervous system (Wang et al., 2020a; Cheng et al., 2021; Sun et al., 2023; He et al., 2024; Sundaram et al., 2024; Khalili et al., 2025). Research has shown that Amuc_1100 attenuates depressive-like behaviors in mice subjected to chronic unpredictable mild stress, demonstrating that it alleviates depressive symptoms by reducing the serum 5-HT levels in mice and increasing BDNF levels in the hippocampus, thus exerting repairing and protective effects on the nervous system (Cheng et al., 2021). Research has shown that the outer membrane protein Amuc_1100 of AKK can directly bind to human TLR2 (hTLR2) *in vitro*, and its dimeric form has a stronger binding capacity to hTLR2 (Wang et al., 2020a). Based on these findings, we can also conclude that the outer membrane protein Amuc_1100 and hTLR2 structural variants may constitute new drug targets. A meta-analysis examined the behavioral effects of Amuc_1100 on anxiety, depression, and stress in mice reported in studies published prior to 2024. The results showed that it can reduce neuro-related biochemical indicators, improve learning and motor abilities, and alleviate depressive-like behaviors (Khalili et al., 2025). Another article discussed the cognitive impairments associated with ageing. A study revealed that in aging mice, L-arginine and the gut microbiota become imbalanced, and a decrease in the proportion of AKK leads to cognitive impairments (He et al., 2024). After supplementation with Amuc_1100, plasma L-arginine levels increase, and Amuc_1100 reduces oxidative stress levels, increases nitric oxide levels, enhances synaptic plasticity, and ultimately improves learning, memory, and synaptic function in aged mice (He et al., 2024). A study has shown that Amuc_1100 can stimulate the secretion of glucagon-like peptide-1 (GLP-1), whose receptors are distributed throughout the nervous system and peripheral organs (Sundaram et al., 2024). Activation of the GLP-1 pathway can promote the release of anti-inflammatory cytokines by microglia, ultimately protecting microglia, blood vessels, and neurons (Sundaram et al., 2024). In addition, experiments with antibiotic-induced depression and anxiety models in mice have shown that Amuc_1100 reduces alterations in cortisol, cellular proto-oncogene fos (c-Fos), and glial fibrillary acidic protein levels. These effects, observed through behavioral assessments and biochemical and protein analyses in some mice, demonstrate that Amuc_1100 may affect brain function and mood through the brain–gut axis (Sun et al., 2023). All of the above studies have explored new fields of neurological disorders and their cognitive functions (Wang et al., 2020a, b; Cheng et al., 2021; Sun et al., 2023; He et al., 2024; Sundaram et al., 2024; Khalili et al., 2025). However, the above studies involve only mouse experiments, and in the future, large-scale clinical trials should be conducted to determine the effects of Amuc_1100 on depression, anxiety, cognitive impairment, and other functions to better evaluate its efficacy and safety in humans (Wang et al., 2020a, b; Chen et al., 2022; Sun et al., 2023; He et al., 2024; Sundaram et al., 2024; Khalili et al., 2025). For example, the article mentioned above appears to focus primarily on mouse models.

Although Amuc_1100 seems to ameliorate cognitive impairment, these findings suggest that it still needs to be tested in human subjects in the future. I believe that these considerations are particularly important within the broader analytical framework of drug development, as we first need to observe whether the compound causes adverse effects on various organs and then conduct small-scale clinical trials to evaluate the safety of Amuc_1100 in humans and select an appropriate dose range. Given the complexity of these methodological considerations, long-term and predominantly large-scale clinical trials will be needed to further evaluate the efficacy of these methods (He et al., 2024). In addition, future human studies should provide more effective methods of administration to improve the therapeutic efficacy of Amuc_1100 and reduce side effects. The above research was also conducted on a mouse model, but the model did not receive oral gavage in the control group (Sun et al., 2023). However, we also need to understand whether gavage causes other stresses and potentially alters other factors in mice (Sun et al., 2023). Previous studies have not explored whether a difference exists in the neurological improvement effect of different doses of Amuc_1100 (Zhu et al., 2024; Jena et al., 2025), and the optimal dosing time has not been investigated. In a recent study, PD-1 immunotherapy was administered to a mouse cancer model along with three forms of AKK: inactivated AKK and the membrane protein Amuc_1100 (Zhu et al., 2024). However, Zhu et al. (2024) focused primarily on a mouse model of lung cancer, and although a bioinformatics analysis was conducted to determine the mechanism of action of AKK in the tumor microenvironment, further experiments are needed to confirm the biological consequences and potential mechanisms. Research has shown that Amuc_1100 can regulate the expression of tight junction proteins and reduce the production of pro-inflammatory factors, thereby altering intestinal barrier function and affecting the absorption of nutrients and the metabolic transport of microorganisms, particularly the expression of SCFA transport proteins. However, its potential for treating neurological diseases remains to be explored (Jena et al., 2025). In the future, single-cell RNA sequencing and metabolomics can be used to elucidate the specific mechanism of Amuc_1100 in neurological diseases.

### Extracellular vesicles

AKK-derived EVs (AKK-EVs) are nanoscale, lipid bilayer-enclosed structures that contain bioactive molecules, including proteins, RNAs, lipids, and sugars, and serve as important mediators of intercellular communication (van Niel et al., 2018; Zhao et al., 2024). Our analysis suggests that these microbial vesicles have attracted widespread research interest because they play multifaceted roles in immune regulation, intestinal barrier maintenance, metabolic regulation, and neuroprotection. Importantly, within this broader analytical framework, EVs possess a unique advantage in their apparent ability to persist in the host for extended periods without seemingly eliciting immune rejection (Díez-Sainz et al., 2022). This characteristic, coupled with their capacity for targeted organ delivery, generally indicates a mechanism that largely minimizes cellular side effects. Given the complexity of these theoretical relationships, the evidence reveals a promising avenue for therapeutic applications that warrants further consideration (Díez-Sainz et al., 2022). These properties render them particularly promising for clinical applications. Current research has focused primarily on their therapeutic potential for treating intestinal inflammation and metabolic disorders (Akbar et al., 2019; Liang et al., 2022). For example, AKK-EVs exert protective effects on colitis models by reducing inflammatory cytokine expression, enhancing intestinal epithelial tight junctions, and modulating the gut microbiota composition by increasing the abundance of beneficial bacteria while suppressing the growth of pathogenic species (Zheng et al., 2023a). Additionally, AKK-EVs exhibit immunomodulatory properties by regulating CD8^+^ T-cell and macrophage activity, with demonstrated antitumour effects on prostate cancer in both *in vitro* and *in vivo* models (Luo et al., 2021).

Emerging research has begun to explore the neuroprotective potential of AKK-EVs, and a recent study has indicated their ability to mitigate postoperative cognitive dysfunction induced by intestinal ischemia‒reperfusion, a condition particularly prevalent in older adult patients (Gao et al., 2024). The therapeutic mechanism involves the inhibition of TLR2/TLR4-mediated inflammatory cascades, including the downregulation of NF-κB activation, which subsequently attenuates microglial inflammatory responses and improves cognitive function. These findings position AKK-EVs as promising therapeutic candidates for treating postoperative cognitive dysfunction (Gao et al., 2024). One study confirmed that AKK-EVs can increase 5-HT levels in the colon and hippocampus of mice, while serum 5-HT levels tend to decrease. Furthermore, no inflammatory cell infiltration was observed in the colons of mice treated with AKK-EVs, confirming their safety (Yaghoubfar et al., 2020). Another study reported that 5-HT is an important neurotransmitter that regulates the brain–gut axis (Yaghoubfar et al., 2023). Most 5-HT is produced in the intestine, and it can regulate brain function through the vagus nerve (Yaghoubfar et al., 2023). Although the study by Yaghoubfar et al. (2023) did not explicitly mention related neurological diseases, AKK and AKK-EVs can influence the expression of 5-HT in the intestine after administration via oral gavage. Therefore, we speculate that this vesicle delivery method can also indirectly affect the nervous system. However, despite these encouraging results, research on the effects of AKK-EVs on neurological function remains in its early stages, with several key knowledge gaps still unresolved. These gaps include the complexity of EV mechanisms, particularly how diverse vesicular components interact to exert therapeutic effects, as well as the heterogeneity of EVs in terms of size, morphology, composition, and function under different physiological states, complicating standardization for therapeutic applications (Chelakkot et al., 2018). Moreover, although AKK-EVs activate AMPK and regulate tight junctions, the specific vesicular component responsible remains unidentified. The distribution ratio of AKK-EVs in intestinal versus peripheral tissues is unknown, and challenges such as understanding EV life cycles and improving *in vivo* delivery efficiency further hinder their therapeutic development (Chelakkot et al., 2018). For example, one study revealed that AKK-EVs can regulate the intestinal endocannabinoid system, and the results indirectly suggested that AKK-EVs are related to mood regulation; however, verification in animal or clinical models is lacking, and their direct regulation of neurotransmitters has not been explored (Noori et al., 2025). Additionally, behavioral verification is lacking (Noori et al., 2025). Further challenges include understanding EV life cycles and optimizing *in vivo* delivery efficiency. A study using EV hybrid lipid nanocarriers for cancer immunotherapy revealed that nanocarriers loaded with plasmids have different cellular uptake rates in various cells, indicating that their delivery efficiency also varies (Tong et al., 2023). For example, one study showed that although AKK-ABs produced effective therapeutic effects on obese models, they also had poor stability, were difficult to produce on a large scale, and triggered some immune responses (Ashrafian et al., 2019). Future research should prioritize mechanistic work to elucidate these aspects, which will be crucial for advancing AKK-EV-based therapies from the bench to the bedside.

### Pasteurized *Akkermansia muciniphila*

Pasteurization, a heat treatment method pioneered by Louis Pasteur, is widely used for microbial inactivation in food and biological samples (Inanoglu et al., 2022). This process involves heating samples to specific temperatures (typically 60–85°C) for several minutes, effectively eliminating pathogenic microorganisms while preserving essential biological components. The antimicrobial mechanism involves the thermal denaturation of microbial proteins, DNA damage, and disruption of cellular membrane integrity (Calahorrano-Moreno et al., 2022). While AKK naturally functions as a probiotic in the gut, its pasteurized form has unique therapeutic advantages (Druart et al., 2021). For example, pasteurized AKK has shown efficacy in regulating lipid metabolism in Caenorhabditis elegans by reducing fat accumulation, inhibiting lipogenesis, and enhancing fatty acid oxidation, effects attributed to reduced oxidative stress and an extended lifespan (Wu et al., 2022). In type 2 diabetes models, the P5 protein isolated from pasteurized AKK activates G protein-coupled receptor signaling to stimulate glucagon-like peptide production, thereby improving glycaemic control (Niu et al., 2024). Comparative studies have revealed that pasteurized AKK tends to outperform live AKK in certain applications, particularly in ulcerative colitis models, where it promotes beneficial microbiota proliferation, substantially increases SCFA production, and seemingly reduces intestinal inflammation (Xue et al., 2023; Han et al., 2025). In addition, a previous study revealed key membrane proteins such as Amuc_1100 in live and pasteurized forms (Han et al., 2025). The results showed that live bacteria may be inhibited by competition from the host flora and that pasteurization is safer and more stable (Han et al., 2025). Another study suggested that a pasteurized AKK intervention can regulate intestinal barrier genes and alter the composition of the intestinal microbiota (Liu et al., 2023). The mice were treated with pasteurized AKK for 2 weeks and then randomly infected with *Salmonella typhi* (Liu et al., 2023). The findings from the study by Liu et al. (2023) suggest that pasteurized AKK has the potential to prevent Salmonella infection, which appears particularly significant within this broader analytical framework. AKK is used to treat preeclampsia-like symptoms, as pasteurized AKK ostensibly retains the main active components of AKK, such as surface proteins and cell wall components (Wang et al., 2025a). Given the complexity of these theoretical relationships, these two AKK formulations can protect metabolism and immunity, and pasteurized AKK appears to effectively reduce the risk of infection caused by live bacteria, thereby providing protection during pregnancy (Wang et al., 2025a).

Pasteurized AKK possesses substantial neuroactive properties through gut–brain axis modulation. In irritable bowel syndrome models, it appears to alleviate abdominal pain and anxiety-like behaviors while seemingly improving cognitive function, suggesting potential advantages over conventional irritable bowel syndrome treatments with limited efficacy and adverse effects (Meynier et al., 2024). The evidence reveals that the therapeutic spectrum of pasteurized AKK extends to neurodegenerative and metabolic disorders, given the complexity of these theoretical relationships, as evidenced by improved spatial memory and reduced anxiety/aggressive behaviours in the majority of zebrafish models of AD and diabetes (Qu et al., 2023a). A particularly significant aspect of these findings is how they constitute a promising avenue for further investigation within this broader analytical framework of microbiome-based neurological interventions (Qu et al., 2023a). However, important translational challenges remain. Zebrafish models, while valuable for preliminary research, differ substantially from humans in the nervous system organization, intestinal ecology, and metabolic pathways. Moreover, the complex pathophysiology of diabetes and AD necessitates a careful consideration of interspecies differences. Future research should prioritize clinical validation in human studies, optimize dosing regimens through systematic animal experiments, and investigate the mechanistic effects of pasteurization on the bioactive components of AKK. Another study used pasteurized AKK loaded with astaxanthin because pasteurized AKK does not destroy intracellular proteins or polysaccharide molecules, making it a novel dietary supplement for targeted nutritional delivery to the intestines (Ma et al., 2025b). Another article identified that PP, the total protein of pasteurized AKK, promotes GLP-1 production and activates the GPCR signalling pathway, and a key protein in PP, P5, ostensibly lowers blood sugar levels (Niu et al., 2024). However, within this broader analytical framework, this article did not fully explore other possible targets of pasteurized AKK, and its long-term effects and safety predominantly remain to be verified through further study, given the complexity of these theoretical relationships (Niu et al., 2024). **Additional Table 2** summarizes the comparative characteristics, therapeutic applications, and challenges associated with different formulations of AKK, providing a framework for future research and clinical translation.

The various derivative forms of AKK each possess distinct characteristics. Compared with live AKK, the outer membrane protein Amuc of AKK, AKK-EVs, and inactivated AKK all exhibit superior efficacy, safety, and stability. An investigation of AKK-EVs indicated that orally administered AKK-EVs can be absorbed by RAW264.7 macrophages. In dextran sulfate soudium (DSS)-induced mice, AKK treatment decreases body weight, alleviates pathological changes in the colon, and increases the expression of tight junction proteins, ultimately maintaining the stability of the intestinal barrier (Zheng et al., 2023a, b). Additionally, after being inactivated by pasteurization at 70°C, AKK maintains the structural integrity and stability of its membrane proteins. (Han et al., 2025). The outer membrane P5 protein mentioned above can also be retained in heat-inactivated AKK, activating GLP-1 secretion through the fatty acid receptor 2 (FFAR2) receptor and ultimately ameliorating T2 diabetes mellitus (Niu et al., 2024). However, live AKK appears to colonize the intestines for only a short period and are easily affected by the intestinal environment. Evidence suggests that pasteurized Pseudomonas aeruginosa substantially upregulates *Muc2* mRNA expression, whereas live Pseudomonas aeruginosa seems to have no obvious effect (Xue et al., 2023). Within this broader analytical framework, prophylactic supplementation with live Bacteroides reduces the levels of the cytokines interferon gamma (IFN-γ), interleukin (IL)-1β, and IL-6 in the majority of cases, whereas pasteurized Bacteroides generally decreases the levels of the cytokines TNF-α, IFN-γ, IL-1β, and IL-6 (Xue et al., 2023). Additionally, supplementation with pasteurized AKK tends to result in higher levels of SCFAs at 14 days than supplementation with live AKK (Xue et al., 2023). Therefore, this pattern suggests that the effect of pasteurized AKK is greater than that of live AKK, given the complexity of these theoretical relationships. In addition, experiments have shown that AKK-EVs are more effective than pasteurized AKK and live bacteria, maintaining stable survival without side effects (Ashrafian et al., 2021a). Furthermore, studies have indicated that live bacteria, pasteurized bacteria, and AKK-EVs significantly reduce TNF-α/IL-6 levels and increase IL-10 levels, but AKK-EVs are more effective (Keshavarz Azizi Raftar et al., 2021). Based on the above research, we can conclude that among the derivatives of AKK, AKK-EVs are the most effective, followed by inactivated AKK, and their efficacy is far greater than that of live AKK.

## Current Status of Clinical Translation

Most existing probiotics originate from Bifidobacterium and Lactobacillus, which are approved by the U.S. FDA. However, with the development of next-generation sequencing technology, novel microorganisms are continuously being discovered and related to health, and the safety, formulation, and administration methods of these new microorganisms are currently under investigation (Iwaza et al., 2022). AKK has many health benefits and has received considerable attention, showing great potential for the future. Regarding the National Institutes of Health’s initiatives on neurological diseases, currently, no clinical human trials are being conducted or reported. However, in recent years, reports concerning obesity and type 2 diabetes have been published. For example, *Cell Metabolism* published the results of a trial (NCT04797442) indicating that oral AKK WST01 is safe and well tolerated, but its metabolism shows individual differences. For patients with initially low levels of AKK in the gut, the treatment has considerable effects, controlling weight and blood sugar levels. However, for those with higher levels of AKK in the gut, the efficacy is limited (Zhang et al., 2025). Additionally, in a study with 32 adult overweight or obese patients who received daily oral doses of 10^9^ or 10^10^ colony-forming units of the live bacteria or inactivated AKK, significant improvements in insulin sensitivity, reductions in fasting insulin levels, slight weight loss, and no changes in the overall gut microbiome structure were detected. These findings indicate that AKK can alleviate the condition of patients with metabolic syndrome (NCT02637115; Depommier et al., 2019). Additionally, related clinical trials conducted in North America (NCT05114018) have explored the relationship between AKK and insulin sensitivity, but the results have not yet been fully disclosed. Although no relevant research has been conducted on AKK in the clinic for nerve damage repair, AKK, a new generation of intestinal probiotics, has great therapeutic potential and human clinical trials must be conducted. Therefore, in the future, we should conduct more clinical trials to prove the therapeutic value of AKK.

## Limitations

Although this article describes the relationship between the ability of AKK to relieve neurological diseases and regeneration in detail, at present, research on AKK is still in its preliminary stage and is insufficient. First, most of the existing studies use animal models or *in vitro* experiments, and large-scale human clinical studies are lacking in terms of their theoretical basis. Second, the specific mechanisms by which AKK regulates nervous system function, such as the molecular pathways by which AKK affects nerve regeneration, neuroinflammation or neuroprotection through the gut–brain axis, are still unclear. Most of the current literature discusses its effects on the nervous system by improving intestinal barrier function and regulating the inflammatory status and metabolites; however, the results have not been verified. Third, strain-specific differences in AKK have been identified. The sources, treatment methods, and administration methods used in different studies vary greatly, which may lead to experimental results that are not of great reference value. Finally, whether AKK has universal relief and therapeutic effects on different types of neurological diseases needs to be further explored in the future. For these restrictive issues, future studies need more in-depth analyses of treatment that combine the advantages and disadvantages of AKK to develop reasonable optimization plans.

## Conclusions and Perspectives

In recent years, research on the regulatory role of AKK in the nervous system through the brain–gut axis has garnered widespread attention. Currently, research has focused mainly on the integrity of the intestinal barrier, the impact on inflammatory factors, the regulation of neurotransmitters, and the effects on neuroinflammation. Although AKK derivatives, specifically outer membrane vesicles, have increased safety and stability, many issues remain in the current research progress. First, most mechanistic studies have been conducted in animal or *in vitro* models and lack sufficient clinical data to support their findings. Second, the exact molecular pathways by which AKK affects the central nervous system are still unclear. For example, can metabolites or membrane proteins cross the blood–brain barrier? While AKK appears to cross the intestinal and blood–brain barriers, what seems especially noteworthy in this analytical context is that the specific mechanisms involved tend to remain poorly understood. Given the complexity of these theoretical relationships, the evidence reveals substantial questions regarding the therapeutic efficacy of AKK across different populations, due to individual variability in the colonization capacity. Importantly, if AKK predominantly causes dysbiosis within the gut environment, it apparently damages gut barrier integrity, exacerbates inflammatory processes, and triggers dysfunction within the central nervous system. Therefore, this pattern suggests that within these evolving conceptual parameters, we typically need to conduct further studies with more nuanced mechanistic investigations to help elucidate the potential clinical applications of AKK in treatment contexts.

AKK, a unique probiotic in the intestine, seems to be sensitive to oxygen and specifically breaks down mucus. Therefore, AKK maintains a dynamic balance, which may explain why it can typically colonize the intestine for a short period. Notably, AKK produces metabolites such as SCFAs and bioactive membrane proteins, which enhance the integrity of the intestinal barrier, regulate host immunity, and modulate signalling in the gut and CNS. These metabolites seem to increase the expression of tight junction proteins and stimulate GLP-1 secretion (Chapela et al., 2025). Additionally, recent studies suggest that AKK alleviates EAE by modulating the gut microbiota, enhancing intestinal barrier function, inhibiting Th17-mediated immune responses, and suppressing NLRP3 inflammation in the hippocampus of mice, ostensibly alleviating neuroinflammation and cognitive impairment (Li et al., 2025). However, the evidence suggests that the therapeutic effects of AKK vary substantially among individuals, which may be due to differences in colonization efficiency and microbial community composition. Based on this analysis, we need to explore individualized therapies in the future.

This review organizes and summarizes the latest advances in our understanding of AKK in the nervous system, highlighting its role in the brain–gut axis. Unlike other reviews on AKK, this one specifically focuses on the nervous system, addressing not only metabolism and anti-inflammatory responses but also further exploring the roles of AKK metabolites, outer membrane proteins, and EVs in regulating neuroinflammation, neurotransmitter metabolism, and improvements in cognitive impairments. These findings provide new targets for future clinical treatments aimed at nerve regeneration and cognitive function recovery. In our review, we compare and summarize the different forms of AKK (such as live bacteria, pasteurized inactivated bacteria, and EVs) in terms of long-term efficacy, safety, and clinical therapeutic effects, suggesting that AKK-EVs may be the most effective treatment form in the future, thereby offering an innovative perspective. Additionally, future research should focus on the therapeutic prospects of AKK in neurodegenerative diseases and inflammatory brain diseases, while clarifying its molecular mechanisms to provide a theoretical basis and research direction for subsequent studies. However, existing research has several shortcomings. First, the therapeutic effects of AKK show remarkable disparities under different intervention conditions. For example, some studies indicate that varying fiber structures can affect the degradation activity of AKK. In one experiment, a randomized, double-blind placebo was used as a control. Subjects were patients with type 2 diabetes who consumed 14 g of cactus powder, 4 g of chia seeds, 30 g of soy protein, and 4 g of inulin for 3 consecutive months, resulting in a 125% increase in AKK levels, indicating that soluble fiber structures can enhance AKK levels (Verhoog et al., 2019). Second, antibiotic pretreatment can also influence the effectiveness of AKK colonization. In mice, antibiotic pretreatment followed by supplementation with live AKK leads to considerably higher levels of AKK mRNA expression in the feces of the supplemented group compared with those who received direct supplementation without pretreatment. These findings suggest that antibiotic treatment remarkably boosts the level of AKK (Qu et al., 2023b). Non-antibiotic treatments can also affect the abundance of AKK. Ke et al. (2021) have shown that administering metformin to a mouse model of ulcerative colitis for 2 weeks remarkably increases the number of goblet cells and enhances the expression of key transcription factors, such as KLF4, SPDEF, and MUC2, thereby increasing the thickness of the mucus layer, which serves as the intestinal barrier. These factors warrant further exploration in the future. In terms of clinical translation as a therapy, AKK produces varying results due to the specificity of its strains, methods of administration, and differences in safety. For instance, the previously mentioned pasteurized AKK still alleviates metabolic syndrome (Plovier et al., 2017). This finding highlights that different preparation methods for strains can impact their final functions. Additionally, a Phase I clinical trial in humans administered live AKK orally to assess safety and tolerance, and after 3 months of supplementation, the levels of liver dysfunction and inflammation-related blood markers decreased, while the structural barrier of the intestines remained unaffected (Depommier et al., 2019). Many novel and promising therapeutic prospects have emerged in current research. For example, a study revealed that the abundance of AKK in the feces of 338 patients with advanced non-small cell lung cancer receiving PD-1 immune checkpoint inhibitors was closely related to treatment efficacy. Moreover, gut symbiotic bacteria similar to AKK were also considerably enriched, suggesting a connection to the tumor microenvironment. These findings indicate that AKK could serve as a biomarker for PD-1 treatment, facilitating stratified therapy for patients (Derosa et al., 2022). Additionally, AKK intervenes in neuroinflammatory conditions and cognitive function impairments (Li et al., 2023; Zhu et al., 2023a).

Although many studies support the beneficial effects of AKK, there are instances where it can produce ‘negative’ results. Research indicates that excessive oral intake of AKK can damage the intestinal mucus layer, reduce the expression of tight junction proteins, impair the intestinal barrier, and exacerbate inflammation and tumor development (Qu et al., 2023b). Several limitations of AKK have been described. Current research on AKK and its derivatives primarily relies on animal models, which may not fully reflect the complexity of human diseases. Therefore, future research should focus on clinical human trials. Additionally, previous studies have shown considerable variability in the strains of AKK used, the dosages administered, and the routes of administration, which can lead to differing impacts on the results. Some proposed mechanisms, such as the specific interactions between AKK derivatives and host neural pathways, remain unverified in humans. Moreover, gaps persist in existing studies regarding precise dose optimization, individual differences in response mechanisms, and the synergistic effects of multiple strains (Yoon et al., 2021; Zhu et al., 2023; Gaifem et al., 2024). Another study identified a novel protein, P9, secreted by AKK; however, its immunogenicity, long-term toxicity, and safety remain to be verified (Yoon et al., 2021). The article also mentioned that whether AKK can colonize the intestine long-term in subjects fed a high-fat diet remains unclear. Zhu et al. (2023b) have reported that AKK can migrate to tumor tissues, regulate the tumor microbiome and metabolic pathways, and exert anticancer effects. Although the experiments utilized metabolomics and 16S rDNA sequencing, Zhu et al. (2023b) did not combine multi-omics sequencing, such as transcriptomics and proteomics. Furthermore, whether AKK entering tumor tissues through the bloodstream causes bacteremia still needs to be studied systematically. A previous study has suggested that the combination of AKK and *Parabacteroides distasonis* may help prevent colitis in mice by enhancing ILC3s and strengthening the intestinal epithelial barrier (Gaifem et al., 2024). This finding suggests that this intervention may promote the proliferation of ILC3s and increase IL-22 expression, potentially improving the integrity of the intestinal mucus layer. However, given the complexity of these theoretical relationships, the synergistic mechanism between the two bacteria remains largely unclear, and whether AKK alone can typically colonize the intestine long-term remains unknown. The potential applicability of this treatment in humans appears particularly noteworthy. Importantly, in this context, AKK may exacerbate disease in the absence of dietary fiber; thus, more individualized assessments of these evolving conceptual parameters are needed (Gaifem et al., 2024). In future studies, it is crucial to confirm the safety and feasibility of AKK by conducting experiments in humans, which poses a challenge. Therefore, numerous relevant clinical trials are needed to confirm the efficacy and safety of AKK. One study suggested that the treatment of antipsychotic-induced metabolic dysfunction with second-generation antipsychotics alters the composition of the gut microbiota, particularly by reducing the abundance of AKK. The evidence indicates that treatment with metformin influences the secretion of the gut microbiota, ostensibly alleviating the condition by affecting metabolic products. However, within this broader analytical framework, most experimental data come from mice, and human clinical trials have not been mentioned. While second-generation antipsychotics are known to reduce AKK levels, whether this reduction is the direct cause of antipsychotic-induced metabolic dysfunction remains unclear (Wang et al., 2021a). Importantly, in a mouse model of acute gouty arthritis, AKK PROBIO, a specific strain of AKK, reduced foot swelling and improved the activity of antioxidant enzymes and levels of inflammatory factors. These findings suggest that AKK PROBIO may alleviate symptoms of arthritis through antioxidant and anti-inflammatory mechanisms. However, given the complexity of these theoretical relationships, these findings have not yet been validated in humans (Ma et al., 2024). Finally, although this review focuses on AKK, the analysis does not provide a detailed comparison with other bacterial species related to the brain–gut axis, which may offer a broader context for assessing its unique or shared functions. Future research should pay attention to these issues for deeper exploration.

Accumulating evidence indicates that the gut microbiota can modulate neural function through immune, metabolic, and neurological pathways. Research has identified AKK as a key regulator of neurodegenerative diseases, mood disorders, and cognitive dysfunction. Therefore, this review provides a comprehensive summary of previous research findings on AKK and its relationship with neural regeneration. The novelty of this review is rooted in the limited number of earlier summary articles on major neurological topics and the absence of summaries detailing how AKK mediates brain–gut axis mechanisms. Therefore, this review thoroughly addresses these aspects. In the future, we recommend that research focus on drug delivery methods and various intervention strategies, as well as explore the full therapeutic potential of AKK by integrating microbiological and neuroscientific approaches, which are essential for addressing current research gaps.

## Additional files:

***Additional Table 1:*** *Summary of AKK and clinically relevant neurological disorders.*

***Additional Table 2:*** *Characteristics, advantages, and disadvantages of different forms of AKK in treating different diseases.*

## Data Availability

*All relevant data are within the paper and its Additional files*.
